# Gender-specific changes of the gut microbiome correlate with tumor development in murine models of pancreatic cancer

**DOI:** 10.1016/j.isci.2023.106841

**Published:** 2023-05-18

**Authors:** Tom Kaune, Heidi Griesmann, Katharina Theuerkorn, Monika Hämmerle, Helmut Laumen, Sebastian Krug, Iris Plumeier, Silke Kahl, Howard Junca, Luiz Gustavo dos Anjos Borges, Patrick Michl, Dietmar H. Pieper, Jonas Rosendahl

**Affiliations:** 1 Department of Internal Medicine I, Martin-Luther-University Halle-Wittenberg, Halle (Saale), Germany; 2 Institute of Pathology, Martin-Luther-University Halle-Wittenberg, Halle (Saale), Germany; 3 Microbial Interactions and Processes Research Group, Helmholtz Centre for Infection Research, Braunschweig, Germany; 4 Klinik für Innere Medizin IV, Universitätsklinikum Heidelberg, Heidelberg, Germany

**Keywords:** Microenvironment, Microbiome, Cancer

## Abstract

Pancreatic ductal adenocarcinoma (PDAC) is a devastating disease with a dismal outcome. To improve understanding of sequential microbiome changes during PDAC development we analyzed mouse models of pancreatic carcinogenesis (KC mice recapitulating pre-invasive PanIN formation, as well as KPC mice recapitulating invasive PDAC) during early tumor development and subsequent tumor progression. Diversity and community composition were analyzed depending on genotype, age, and gender. Both mouse models demonstrated concordant abundance changes of several genera influenced by one or more of the investigated factors. Abundance was significantly impacted by gender, highlighting the need to further elucidate the impact of gender differences. The findings underline the importance of the microbiome in PDAC development and indicate that microbiological screening of patients at risk and targeting the microbiome in PDAC development may be feasible in future.

## Highlights

• *In vivo* pancreatic cancer models allowed detailed analysis of microbiota changes
• Genotype, age, and gender influenced microbiota composition and diversity
• Sequential changes of the gut microbiome in both pancreatic cancer models were revealed
• Detailed gender-specific changes have been identified

## Graphical abstract

**Figure:**
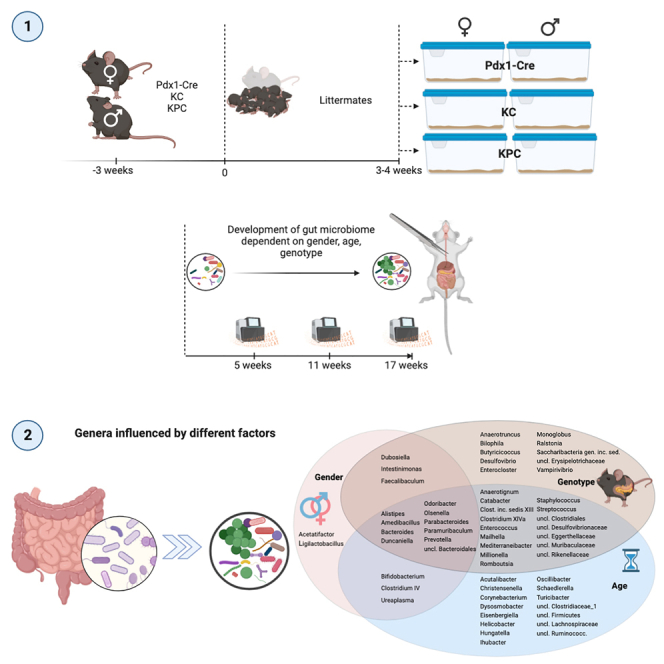

## Introduction

The outcome of pancreatic cancer patients is still dismal and pancreatic ductal adenocarcinoma (PDAC) is expected to be the second leading cause of cancer-related deaths in the United States by 2030.^1^ Further understanding of mechanisms contributing to PDAC development is needed to facilitate the development of novel screening tools and to improve therapeutic approaches. The risk to develop PDAC has been related to several risk factors such as obesity, chronic pancreatitis and smoking. In addition, alterations of the microbiome were reported to induce and modulate the disease or influence progression.^2^^,^^3^

In human PDAC, changes of the oral and also the fecal microbiota correlated with tumor progression.^4^^,^^5^^,^^6^ Of interest, fecal microbiota signatures as a non-invasive diagnostic tool demonstrated an intriguing prognostic capacity to detect PDAC in humans in two independent cohorts.^7^^,^^8^ Also, the pancreas itself was shown to harbor its own microbiota.^9^ Recently it was demonstrated that the intratumoral microbiota of PDAC long-term survivors had a higher a-diversity compared to short-term survivors and a characteristic microbiome signature was prognostic for longer survival.^10^ Transplantation of the respective human fecal microbiota into mice showed its capability to modulate tumor progression, associated with altered recruitment and activation patterns of CD8^+^T-cells in the tumor microenvironment as possible mediator.

In line with these human data, several studies used murine *in vivo* models to improve the understanding of underlying PDAC mechanisms. For example, one of the oral pathogens associated with PDAC, *Porphyromonas gingivalis,* was demonstrated to accelerate the development of pancreatic intraepithelial neoplasia (PanIN) lesions in the established KC mouse model, most likely via enhancing transforming growth factor-β (TGF- ß) signaling.^11^

Distinct microbiome signatures have been shown to alter the immunogenic tumor microenvironment by influencing M1 macrophage and TH1 differentiation as well as CD8^+^T cell activation.^12^ This implies that certain immunophenotypes can be generated by therapeutic intervention in the microbiome. It is important to note that nearly all available studies so far have investigated microbiota changes in patients already diagnosed with PDAC or in tumor-bearing mice, recapitulating an advanced stage of the disease. Subsequently, the identified microbiome signatures were then used for *in vitro* and *in vivo* modeling to demonstrate that the observed changes indeed influence tumor progression. However, these findings only partially reflect the proposed disease continuum from preneoplastic lesions to cancer.

In this context, changes of gut microbiota networks in the adenoma-to-carcinoma sequence observed in colorectal cancer highlight the importance to further elucidate this dynamic process that might be the basis for an early diagnostic tool in the future.^13^ Recent studies have investigated the microbiota in potentially preneoplastic cystic lesions of the pancreas.^14^^,^^15^ Of interest, intra-cystic bacterial 16S rRNA gene content differed significantly between intraductal papillary mucinous neoplasm (IPMN), mucinous cystic neoplasms and serous cystic neoplasms, specifically increasing in IPMN with higher grade of dysplasia where also oral taxa were enriched.^14^ This observation underscores the need to elucidate the role of microbiota during the progression from pancreatic precursor lesions to PDAC.

For the *in vivo* analysis of pancreatic cancer development, the established KC model harboring mutated Kras which recapitulates PanIN progression, and the KPC model with mutated Kras and Trp53 frequently developing invasive PDAC are widely used.^16^^,^^17^ Both complimentary models offer the opportunity to improve the understanding of the dynamic process of early carcinogenesis as well as later cancer development and progression. In addition to the impact of these genotypes, the temporal dynamics and potential gender effects in both models were examined to identify microbiome signatures contributing to cancer development and possible age- and gender-dependent confounders.

## Results

In total, 191 fecal samples of the three genotypes (Pdx1-Cre, n = 17; KC, n = 20; KPC, n = 28) collected at the three indicated time points (5, 11, 17 weeks) were analyzed. Following sequencing and rarefying of library sizes, 1560 phylotypes were observed. The phylotypes belonged to 99 genera and 10 phyla, with sequences of *Firmicutes, Bacteroidetes, Proteobacteria, Actinobacteria*, and *Deferribacteres* comprising approximately 99% of the total bacterial community (Tables S1 and S2).

### Similarities in the bacterial community assemblages

**Figure 1:**
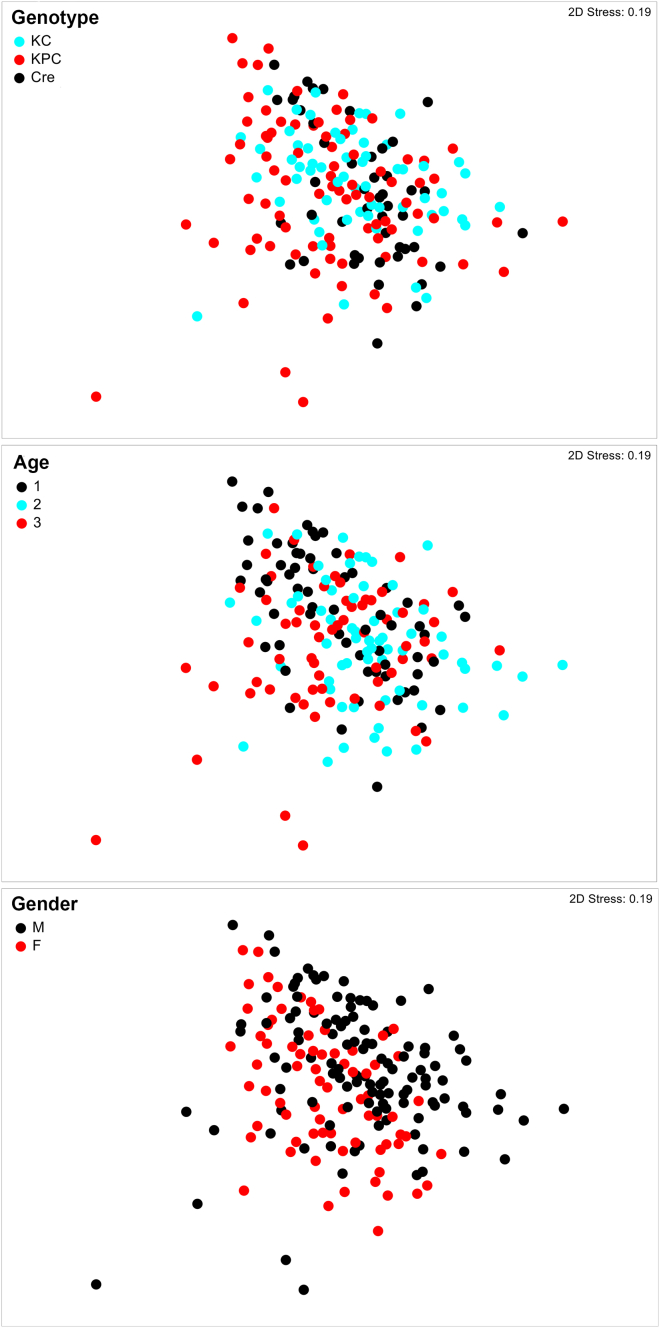
- Differences in global bacterial community structures In Pdx1-Cre, KC and KPC mice of 5, 11 and 17 weeks of age (or date of death see Table S4) the global bacterial community structure was assessed by non-metric multidimensional scaling (nMDS). The global community structure is based on standardized genus abundance data and similarities were calculated using the Bray–Curtis similarity algorithm. The community structures influenced by genotype, age (1 = 5 weeks, 2 = 11 weeks and 3 = 17 weeks) and gender are indicated. Gender: M = male, F = female.

Formal comparisons between the global bacterial assemblages were performed using a three-way design to analyze the influence of the factors mouse genotype (Pdx1-Cre versus KC versus KPC), age and gender. PERMANOVA revealed that all three factors had a statistically significant influence on the community structure on the phylotype level (genotype, pseudo-F = 6.52, p = 0.001; gender, pseudo-F = 6.26, p = 0.001; age, pseudo-F = 4.53, p = 0.001). This difference retained significance in all three parameters at low taxonomic ranks up to the family level. At higher taxonomic ranks, only age had a significant impact (Table S3). Moreover, a statistically significant interaction in the effects of genotype and gender was observed (up to the genus level). Otherwise, a clear interaction was seen in the effects of genotype and age up to the class level. These significant effects are also indicated in the non-metric multidimensional scaling (nMDS) plot (Figure 1).

Pairwise tests revealed that the differences between Pdx1-Cre versus KC or KPC mice were more pronounced compared to differences between KC versus KPC mice (Table 1). During aging the most striking changes were observed between mice of 5 and 11 weeks of age whereas further changes in older mice remained significant but were less pronounced.

**Table 1:** Factors influencing global community structures as indicated by PERMANOVA

Factor	Groups compared	Phylotype		Genus		Family		Order		Class		Phylum	
		t	p(MC)	t	p(MC)	t	p(MC)	t	P(MC)	t	p(MC)	t	p(MC)
Genotype	KC, KPC	1.70	**0.003**	1.66	**0.018**	1.54	0.045	1.04	0.325	1.03	0.335	0.43	0.844
	KC, Cre	2.73	**0.001**	2.38	**0.001**	2.28	**0.006**	1.68	0.054	1.69	**0.040**	0.65	0.666
	KPC, Cre	3.12	**0.001**	2.71	**0.001**	2.60	**0.002**	1.33	0.146	1.34	0.156	0.79	0.502
Age	1, 2	2.23	**0.001**	2.67	**0.001**	2.91	**0.001**	3.27	**0.001**	3.28	**0.001**	4.86	**0.001**
	1, 3	2.52	**0.001**	2.50	**0.001**	2.60	**0.001**	2.91	**0.002**	2.91	**0.002**	4.14	**0.001**
	2, 3	1.48	**0.011**	1.80	**0.009**	1.86	**0.011**	1.30	0.158	1.31	0.146	0.84	0.504
Gender	M, F	2.50	**0.001**	2.62	**0.001**	2.55	**0.001**	1.64	**0.043**	1.60	0.053	1.24	0.202

The influence of genotype, age and gender on microbial structures was calculated by PERMANOVA (pairwise test). The t statistics and the Monte Carlo pvalues are given for each pair of groups of the factors performed at different taxonomic levels (from phylotype to phylum). Age: 1 = 5 weeks, 2 = 11 weeks and 3 = 17 weeks (or date of death); gender: M = male, F = female.

Pairwise analysis of the interactions between the different factors revealed differences between female and male mice. For example, the communities were significantly different on the genus and family level in male KC and KPC mice only (Table S5). Moreover, assemblages in Pdx1-Cre versus KC or KPC mice were significantly different at all analyzed ages, including 5 weeks of age when morphological differences within the pancreas were minimal. Assemblages between KC versus KPC mice were significantly different only after 17 weeks (Table S5).

### The diversity of fecal communities depends on genotype, age and gender

Further differences in the microbial communities were indicated by one-way analysis of α-diversity measures (Tables S6 and S7). Phylotype richness was significantly higher in Pdx1-Cre compared to KPC mice (562 ± 49 versus 533 ± 83 phylotypes, p = 0.0391), with KC mice communities adopting mean values (544 ± 59). Also, older mice showed a higher phylotype richness compared to younger animals (554 ± 82 versus 529 ± 60 phylotypes, p = 0.036) and females had a higher phylotype richness than male mice (557 ± 52 versus 536 ± 77 phylotypes, p = 0.0224). Significant differences in diversity were observed between female and male mice (Shannon index H= 4.301 ± 0.28 versus 4.192 ± 0.366, p = 0.0207; Simpsons index 1-**λ** = 0.9711 ± 0.014 versus 0.9580 ± 0.021, p = 0.0363 (Table S6 and Figure S1).

**Figure 2:**
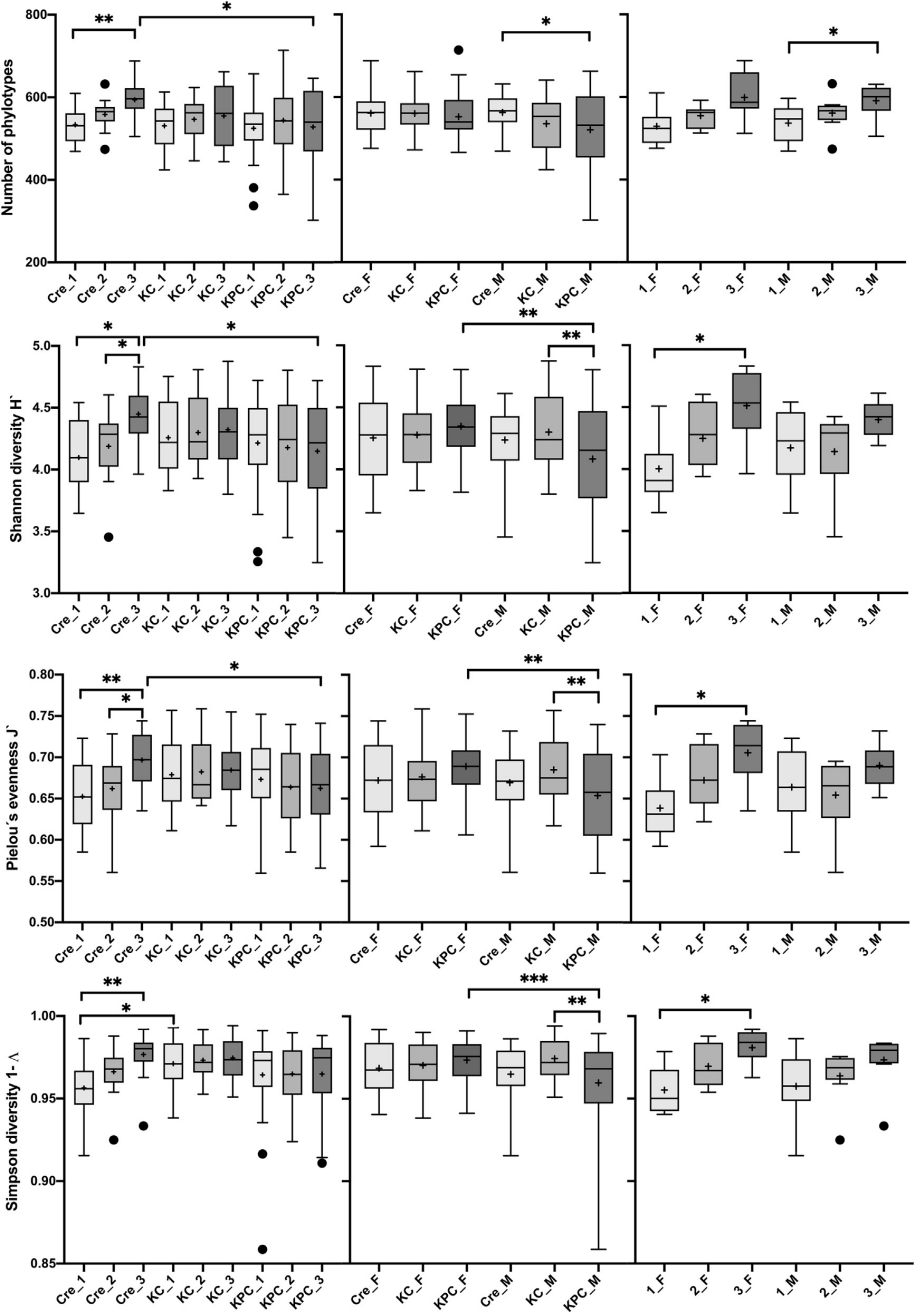
- Fecal bacterial community diversity Diversity is indicated (from top to bottom) by total phylotype number, Shannon diversity (H′), Pielou’s evenness (J′) and Simpsons diversity (1-**λ**), respectively. Analysis was performed by two-way ANOVA analysis using genotype (left), genotype and gender (middle) or age and gender (left) as factors (see also Tables S8–S10 for detailed data). Statistically significant differences between animals with the same genotype (Pdx1-Cre, KC and KPC) but different age (1 = 5 weeks, 2 = 11 weeks and 3 = 17 weeks), or of animals with the same age, but distinct genotypes is indicated on the left. Statistically significant differences between animals of distinct gender (M, F) of the genotypes (Pdx1-Cre, KC or KPC) are indicated in the middle. Statistically significant differences between animals of different age (1 = 5 weeks, 2 = 11 weeks or 3 = 17 weeks) and gender (M, F) of Pdx1-Cre mice are indicated on the right. Significance is shown as ∗p<0.05 or ∗∗p<0.01. The mean is indicated by + and the median by a black line. The box represents the interquartile range. The whiskers extend to the upper adjacent value (largest value = 75^th^ percentile +1.5 x IQR) and the lower adjacent value (lowest value = 25^th^ percentile - 1.5 x IQR) and dots represent outliers. Gender: M = male, F = female.

More detailed differentiations could be obtained through two-way ANOVA analyses, which showed that diversity increased with age in Pdx1-Cre but not in KC or KPC mice (Figure 2 and Table S8) such that 17-week-old Pdx1-Cre mice exhibited a significantly higher diversity compared to KC or KPC mice at the same age.

In addition, two-way analysis revealed that whereas in female mice, the fecal communities of different genotypes did not differ significantly in diversity, specifically male KPC mice showed a rather low diversity, which was significantly lower than that of female mice (Figure 2 and Table S9). As diversity increased with age in Pdx1-Cre mice, it was also analyzed if this increase was influenced by gender. As shown in Figure 2 and Table S10 female Pdx1-Cre mice showed a significant increase in fecal microbial community diversity (Shannon and Simpsons index) and evenness and a trend in phylotype richness (p = 0.066) with increasing age whereas a significant increase in phylotype richness only was visible in male Pdx1-Cre mice.

### The microbial community composition depends on genotype, age and gender

Differences in microbial community composition depending on the different genotypes, age and gender were initially evaluated independently by one-way analyses. Out of the 99 identified genera or genus level taxa (Table S2), 74 were observed with a prevalence of >20% in at least one subgroup and thus subject to a differential abundance analysis. Overall, 59 out of 74 genera (79.7%) were influenced by at least one of these factors as summarized in Figure S2.

**Figure 3:**
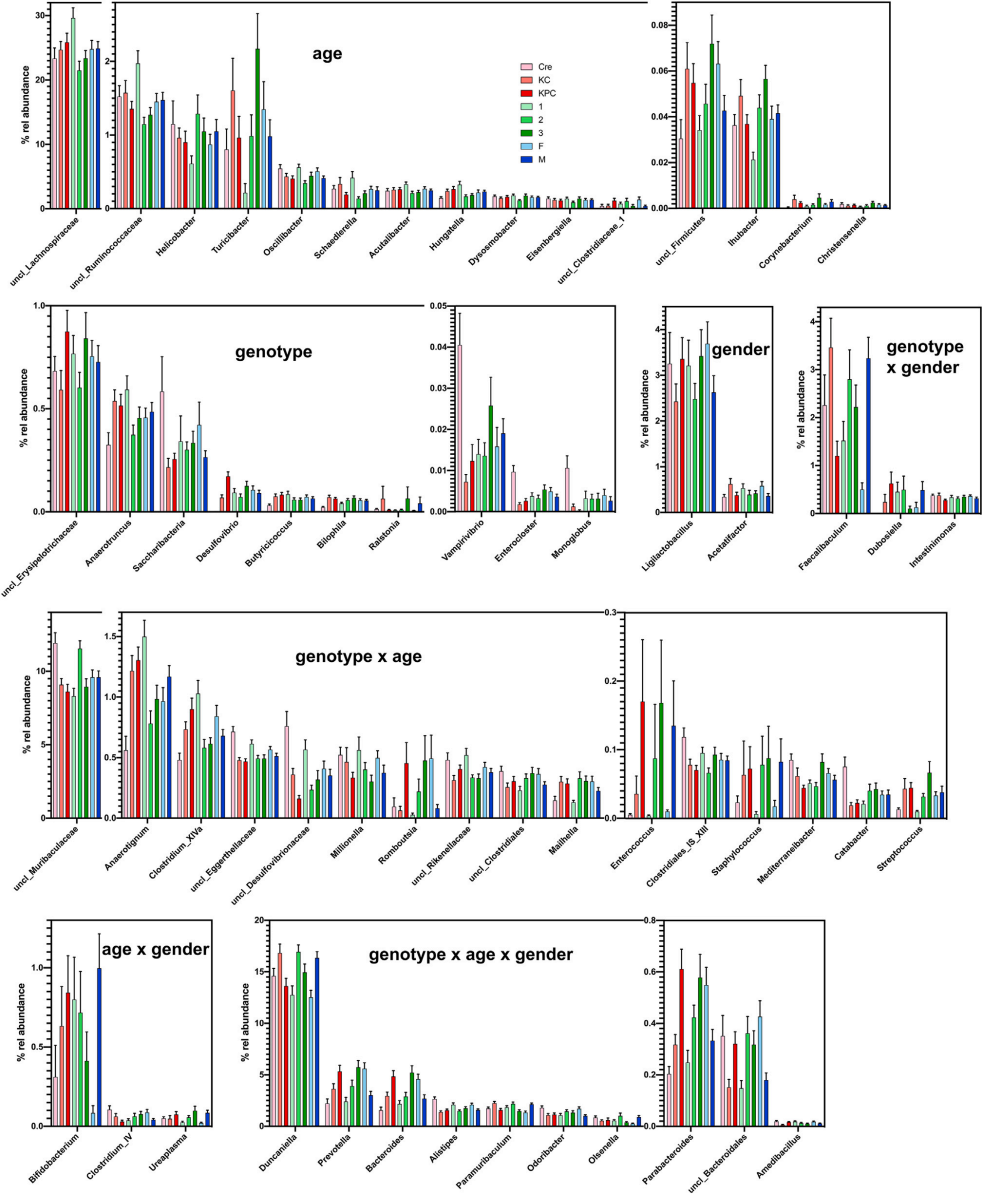
Relative mean abundance of genera and genus level taxa The mean relative abundance of genera and genus level taxa (Table S2) dependent on the genotype (light red, Pdx1-Cre mice; medium red KC mice; dark red KPC mice), age (light green, 5 weeks; medium green, 11 weeks; dark green, 17 weeks) and gender (light blue, female; dark blue, male mice) is displayed as well as the standard error of mean. For each taxon, eight mean relative abundances are therefore shown. Differences in taxon distribution were evaluated independently for each factor by one-way analyses using the Kruskal-Wallis test (factor genotype), the Friedmann test (factor age) or the Mann-Whitney test (factor gender). pvalues are compiled in Tables S11, S12, and S13. The taxa are sorted according to the factors influencing their abundance, which are indicated in the figure.

Out of the 15 genera and genus level taxa mainly influenced by age, the strongest effect was observed for *Turicibacter*, where the mean abundance increased by one order of magnitude (Figure 3 and Table S11). Ten genera and genus level taxa were mainly influenced by genotype (Figure 3 and Table S12). The most pronounced effects were observed for *Monoglobus*, which was practically absent from KPC mice and showed roughly one order of magnitude lower abundance in KC compared to Pdx1-Cre mice. Likewise, *Enterocloster, Vampirovibrio* and *Saccharibacteria* were all found in Pdx1-Cre mice in a mean abundance at least twice or thrice that of KC or KPC mice. Genera influenced by gender were scarce. *Ligilactobacillus* and *Acetatifactor* seemed to be influenced by gender only, being slightly more abundant in female mice. However, for some genera that were also influenced by genotype such as *Faecalibaculum* (6-fold higher mean abundance in male mice) or by age such as *Bifidobacterium* (more than 10-fold higher mean abundance in male mice), marked gender influences were visible. Overall, 18 genera and genus level taxa were significantly influenced by gender (Figure 3 and Table S13). Fifteen genera and genus level taxa, respectively, were influenced by age and genotype and 10 were affected by all three factors. For example, three Bacteroidales genera, i.e., *Bacteroides*, *Prevotella*, and *Parabacteroides,* showed similar effects with increasing abundances both with age and from Pdx1-Cre over KC to KPC mice, with higher levels in females compared to male mice.

**Figure 4:**
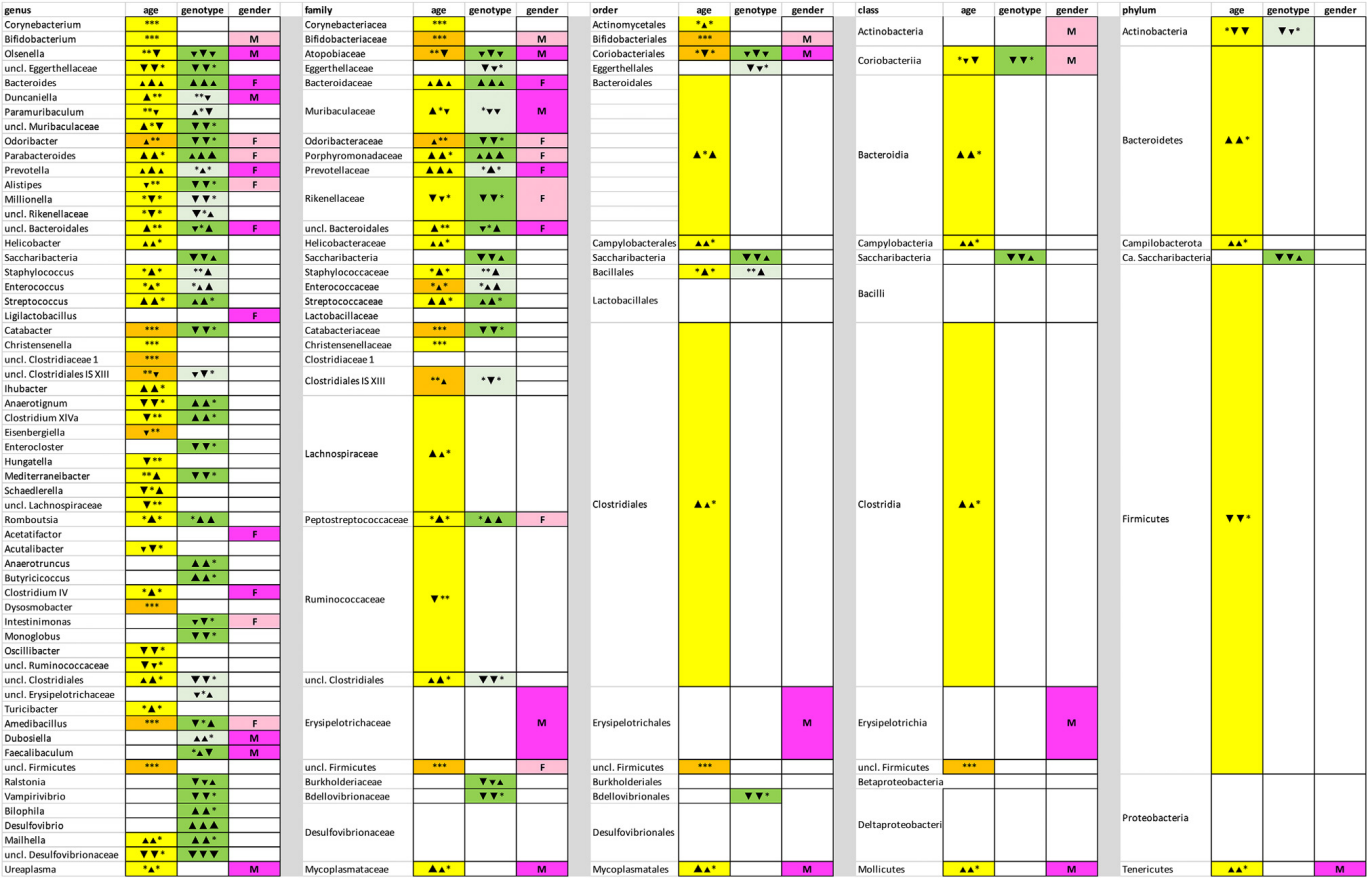
Phylogenetic taxa (genera, families, orders, classes and phyla) influenced by genotype, age and gender Relative abundance date dependent on age, genotype and gender were used (Table S2) and differences in taxon distribution were evaluated independently for each factor by one-way analyses using the Kruskal-Wallis test (factor genotype), the Friedman test (factor age) or the Mann-Whitney test (factor gender) (Tables S11, S12, and S13). Factors significantly influencing the relative abundance of a given taxon are indicated in yellow (age), dark green (genotype) or magenta (gender) if p <0.01 and by orange (age), light green (genotype) or light magenta (gender) if p = 0.01–0.05. A significant increase (p<0.01) with age or from Pdx1-Cre over KC to KPC mice is indicated by a large ▲ symbol, a decrease by a large ▼ symbol. A significant increase/decrease with p = 0.01–0.05 is indicated by the small symbols ▲ or ▼. The comparisons given for each factor and for each taxon are 5 weeks/11 weeks, 5 weeks/17 weeks and 11 weeks/17 weeks and Pdx1-Cre/KC, Pdx1-Cre/KPC and KC/KPC, respectively. In case of gender, the gender with the higher abundance is indicated as F (female) or M (male).

A phylogenetic analysis indicated that *Bacteroidales* were typically affected by all three factors and Erysipelotrichiaceae were prone to be influenced by gender (Figure S3). In contrast, Proteobacteria were mainly influenced by genotype (Figures 3 and S3). Various *Clostridiales* genera were influenced by both genotype and age. However, whereas for example *Anaerotruncus* and *Anaerotignum* of the Ruminococcaceae and Lachnospiraceae were of lower abundance in Pdx1-Cre mice, *Monoglobus* and *Enterocloster* from the same families exhibited a higher abundance in Pdx1-Cre mice. Therefore, the influence of the genotype was clearly observed at the genus level, but to a lesser extent at higher phylogenetic levels (Figure 4). Similarly, genotype effects were observed for various genera and families inside the *Bacteroidales*, but not at class level for the *Bacteroidales* itself (Figure 4).

**Figure 5:**
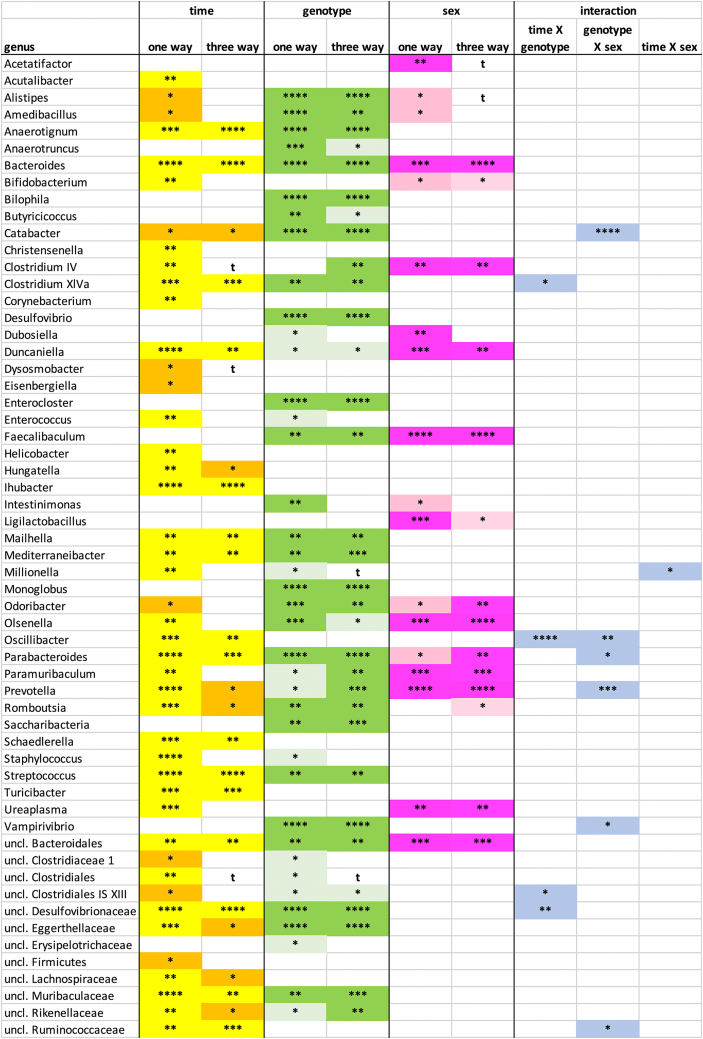
Genera influenced by genotype, age and gender as indicated by one-way and three-way analyses Relative abundance date dependent on age, genotype and gender were used (Table S2) and differences in taxon distribution were evaluated independently for each factor by one-way analyses using the Kruskal-Wallis test (factor genotype), the Friedmann test (factor age) or the Mann-Whitney test (factor gender) (Tables S11, S12, and S13). Differences were also evaluated by a three-way ANOVA using square root transformed relative abundance data. Factors influencing significantly the relative abundance are indicated in yellow (age), green (genotype) or magenta (gender). ∗, p<0.05; ∗∗, p<0.01; ∗∗∗, p<0.001; ∗∗∗∗, p<0.0001; t (trend), p<0.1. Possible significant interactions between factors were evaluated by three-way analysis.

As possible interactions between the different factors could be expected, we performed three-way ANOVA analysis on the genus level. Here, the results of the one-way analysis could be largely confirmed. In 44 of the 46 cases (96%) where a comparison by one of the methods had indicated a significant difference in abundance to be caused by one factor with p<0.001, the second method also indicated a significant influence. In addition, in 73 of the 90 cases (81%) where a significant influence of one of the factors with p<0.01 was observed by one method, the second method gave concordant results (Figure 5).

**Figure 6:**
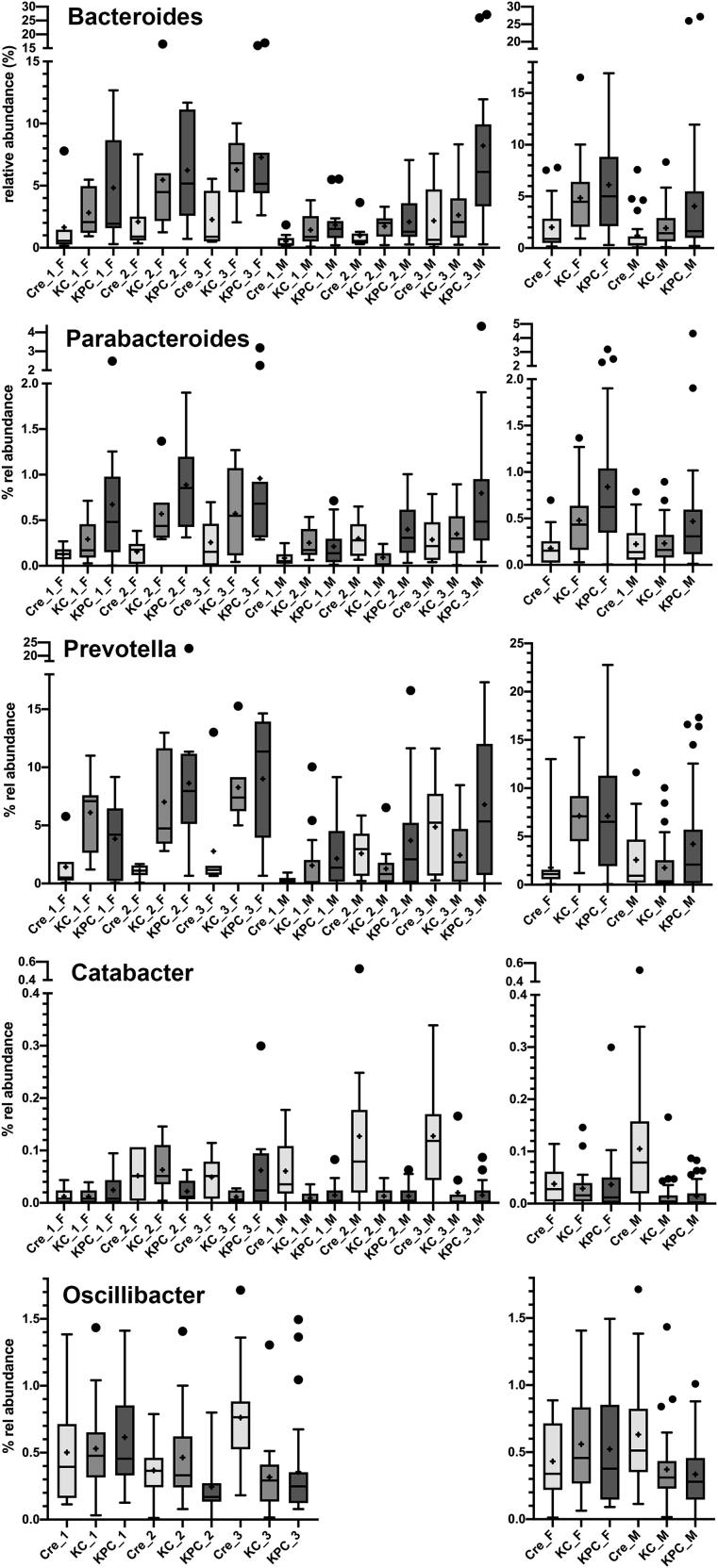
Relative abundance of *Bacteroides*, *Parabacteroides*, *Prevotella, Catabacter* and *Oscillibacter* as influenced by genotype, age and gender The left graph (except for *Oscillibacter*) shows relative abundances separately for each genotype, age and gender (Table S2). In case of *Oscillibacter*, the left graph shows the relative abundances for the different genotypes and gender overall ages. The right graph always shows the relative abundances for the different genotypes and gender overall ages. The mean is indicated by + and the median by a black line. The box represents the interquartile range. The whiskers extend to the upper adjacent value (largest value = 75^th^ percentile +1.5 x IQR) and the lower adjacent value (lowest value = 25^th^ percentile - 1.5 x IQR) and dots represent outliers. F, female; M, male.

In a few cases, a significant interaction between factors was observed. For *Bacteroides* all three factors significantly influenced its relative abundance (Figure 5). The abundance increased from Pdx1-Cre over KC to KPC mice at nearly all time points and in both males and females (Figure 6). Interactions were observed for *Parabacteroides* and specifically *Prevotella* between genotype and gender (Figure 6). There was a pronounced effect of genotype on the abundance of *Parabacteroides* in female, however, only a poor effect in male mice. In case of *Prevotella*, a higher abundance was observed in female KC and KPC compared to Pdx1-Cre mice, whereas no such effect was visible in male mice.

A strong interaction was also observed between gender and genotype for the abundance of *Catabacter*. Whereas female mice of the different genotypes harbored similar abundances, the abundance of this genus was dramatically higher in male Pdx1-Cre compared to male KC or KPC mice. In the *Oscillibacter* genus strong interactions were visible between genotype and gender but even more drastically between genotype and age. As shown in Figure 6 only male but not female Pdx1-Cre mice showed higher abundances compared to KC or KPC animals and only in aged mice an effect of the genotype was visible.

### Differentially distributed species level taxa

Three-way analyses have also been performed to evaluate significant differences in distribution of species level taxa. This has been done specifically to distinguish between different *Bacteroides*, *Duncaniella, Parabacteroides* and *Prevotella* species, as well as between different groups of unclassified Eggerthellaceae, unclassified Lachnospiraceae, unclassified Muribaculaceae and unclassified Ruminococcaceae (see Figure S4). In case of the *Bacteroides* genus, nine of the clusters of similar sequence were observed in >20% of the samples and most of them were influenced by age, genotype and gender, with increasing abundance with age, increasing abundance from Pdx1-Cre over KC to KPC mice and a higher abundance in female mice indicating that most *Bacteroides* spp. behaved similarly. *Parabacteroides distasonis* showed a very similar behavior (Figure S5 and Table S14).

Generally, only in a few cases the 16S rRNA gene sequence gave an indication of the harboring species and frequently, closely related isolates of a defined species were not available. For example, 15 prevalent sequence types indicating the presence of different *Duncaniella* species were observed. These bacteria, with only three species being available so far (*Duncaniella muris*, *Duncaniella dubosii*, *Duncaniella freteri*), exhibited clearly different behavior regarding abundance depending on gender, genotype and age. Different behavior was also observed for *Anaerotignum* sequence types. Obviously, different species of these genera have very distinct characteristics enabling them to react differently to environmental changes.

**Figure 7:**
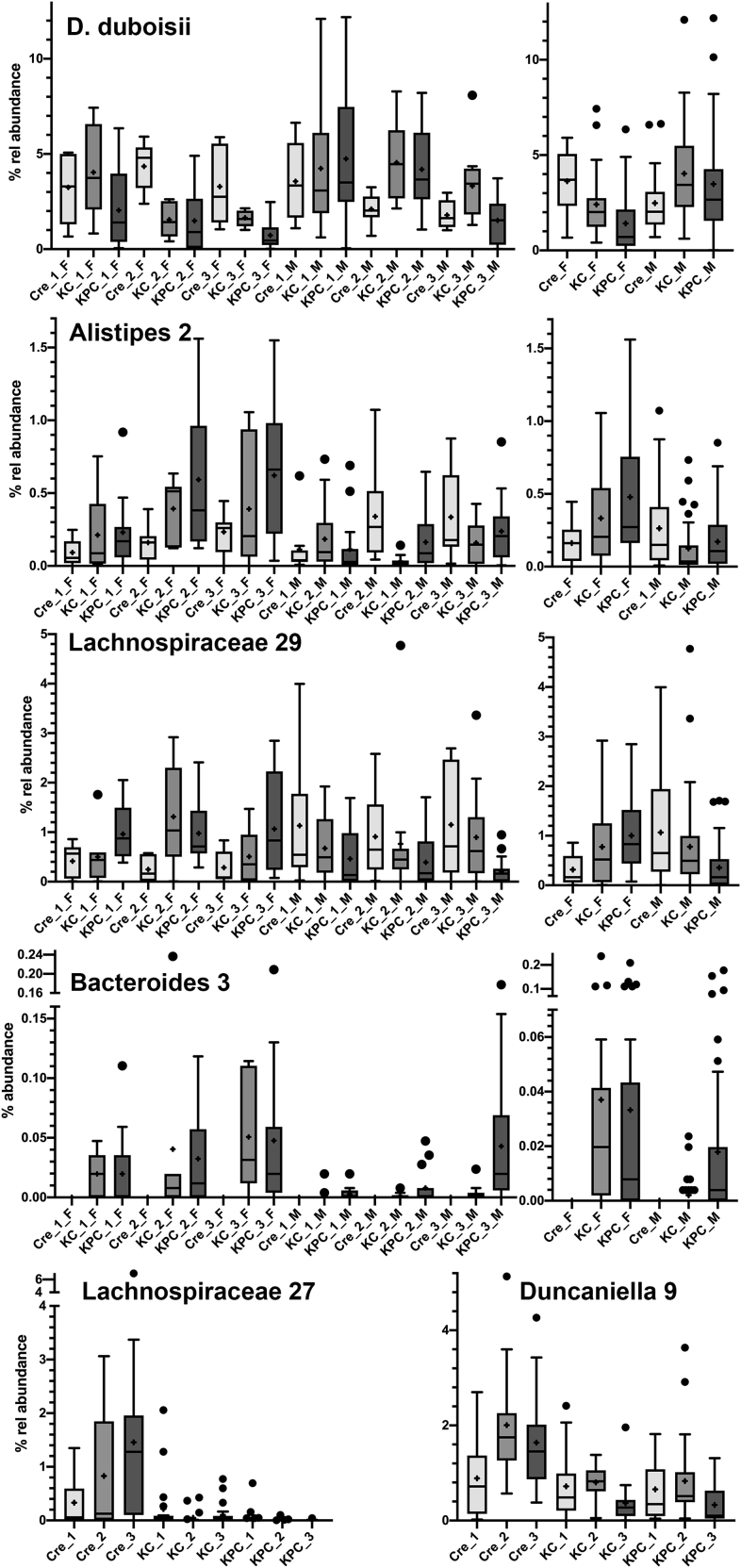
Relative abundance of *Duncaniella duboisii*, *Alistipes 2*, Lachnospiraceae 29, *Bacteroides* 3, Lachnospiraceae *27* and *Duncaniella 9* as influenced by genotype, age and gender For *Duncaniella duboisii*, *Alistipes 2*, Lachnospiraceae 29, *Bacteroides* 3, the left graph shows abundances separately for each genotype, age and gender, whereas the right graph shows the relative abundances for the different genotypes and gender overall ages (Tables S2 and S14). For Lachnospiraceae *27* and *Duncaniella 9* the graphs show the relative abundances for the different genotypes and gender overall ages. The mean is indicated by + and the median by a black line. The box represents the interquartile range. The whiskers extend to the upper adjacent value (largest value = 75^th^ percentile +1.5 x IQR) and the lower adjacent value (lowest value = 25^th^ percentile - 1.5 x IQR) and dots represent outliers. F, female; M, male.

Similar observations were made for bacterial families, which could not be further classified to the genus or species level. A distinct behavior was evident for the 36 clusters of the unclassified Lachnospiraceae regarding their response to age and genotype. However, only a minority (three clusters) was influenced by gender. This contrasted with *Bacteroidales* for which various clusters were affected by gender. Analysis on the species level gave further insights into interactions between the analyzed factors, specifically on differences in gender-dependent genotype effects. Such gender-specific effects were observed for several of the analyzed taxa (Figure 7).

*Duncaniella duboisii* belongs to the most abundant species observed here, with a mean abundance of 3%. In female mice its abundance decreased significantly from Pdx1-Cre over KC to KPC mice. However, this was not the case in male KC and KPC mice that showed an increased abundance. Similarly, a pronounced genotype effect was seen in female mice in which *Alistipes* 2 increased significantly, in contrast to male mice. Even more strikingly the abundance of Lachnospiraceae 29 increased from Pdx1-Cre over KC to KPC in female, but decreased in male mice. Of note, *Bacteroides* 3 was absent from all Pdx1-Cre animals with a reasonable abundance in KPC animals and in female KC animals only. Various species level taxa showed an interaction between the effects of genotype and age or between gender and age (Figure 7). As an example, Lachnospiraceae 27 bacteria were observed with a high abundance in Pdx1-Cre mice only with an age-dependent increase. *Duncaniella* 9 showed a constant abundance with age in Pdx1-Cre mice but decreased in abundance with age in KC and KPC mice.

## Discussion

Perturbations of the microbiome have in various cases been associated with cancer development or various non-malignant diseases, but it often remains unclear whether changes of the microbiome are the cause or consequence of the disease.^18^ In PDAC, most of the published studies investigated alterations of the microbiome in patients with established cancers, whereas data on the microbiome in patients with preinvasive pancreatic precursor lesions are scarce.^10^^,^^12^^,^^19^ Microbiota associated with PDAC development were investigated *in vitro* and *in vivo* and potentially relevant mechanisms for PDAC development were elucidated. The current study used both the slowly progressing murine KC mouse model as well as the rapidly progressing KPC model of pancreatic cancer and distinct changes of the fecal microbiome in a genotype (control vs. KC vs. KPC), age, and gender dependent manner, strikingly preceding clinical symptoms or pronounced morphological changes in the pancreas could be revealed.

An important observation was that microbiota composition developed in a similar fashion and sequentially in both models despite their substantially distinct PDAC development with more pronounced effects in KPC mice. However, also differences in microbiota development were visible reflecting these differences in the models. Of interest, cancer-associated changes were – at least in part – strongly affected by gender. As an example, whereas the abundance of *Bacteroides* increased from Pdx1-Cre to KC or KPC both in female and male mice, *Parabacteroides* and *Prevotella* increased in females only. So far, it seems that such gender effects have not been considered sufficiently in studies of cancer development and only limited data are available. However, it has been shown that the commensal microbial community alters sex hormone levels and regulates autoimmune disease fate.^20^ Furthermore, it is becoming more and more evident that the microbiota differs between genders, both in animal models and in humans, and that these differences often lead to gender-dependent changes in local inflammation, systemic immunity and susceptibility to a range of inflammatory diseases.^21^

Specifically, *Bacteroides* and related organisms are often reported to be differently distributed between males and females, however, results are sometimes contradictory. In a cross-sectional study from four European countries, gender effects were observed for the *Bacteroides-Prevotella* group, with higher levels in males than in females.^22^ Contrary, others reported a higher abundance of Bacteroides in females^23^ and Haro et al.^24^ showed a lower abundance in males with a high body mass index. In mice, *P. distasonis* was enriched in B6 females compared to B6 males.^25^

*Bacteroides* and *Parabacteroides* also represented key-genera of gender-specific dysbiosis in BTBR T + Itpr3tf/J mice used as autism model. Both genera were significantly more abundant in female and male BTBR mice compared to controls, but abundance differences were more pronounced in females.^26^ This is in line with the results of our study where both genera were specifically enriched in females. Of interest, at least for *Prevotella*, which were enriched in female mice in our study, such enrichment has been associated with several inflammatory diseases.^27^ According to the current data, specific genera seem to have gender-specific effects in PDAC development and this observation needs to be accounted for in future studies. Whether this is also of importance in the human situation remains to be elucidated.

Another finding was the sequential change of abundances with age of various genera in both cancer models. This observation indicated that changes of the microbiome may even be found in the preneoplastic state represented by precursor PanIN lesions in the KC model at ages well before PDAC or advanced lesions developed.^16^^,^^28^ Therefore, it seems reasonable to assume that changes of the microbiome occur in a continuum like in colorectal cancer patients where sequential changes of the microbiome correlated with distinct disease stages.^29^ This offers opportunities for screening of microbiota signatures to detect predisposing factors associated with PDAC development in cohorts with increased PDAC risk, such as patients with mucinous cystic lesions, new-onset diabetes or genetic cancer predispositions. The feasibility of screening for fecal microbiota signatures was recently demonstrated by large multi-center studies in Asian and European cohorts.^7^^,^^8^ In two independent investigations, areas under the curve to predict PDAC ranged from 0.75–0.84. So far, the predictive potential of these signatures might not be sufficient to stand alone but needs to be applied in conjunction with other factors such as exposome data, clinical parameters and/or circulating biomarkers.

As retrograde migration of microbiota from the duodenum to the pancreas is a potential access route, a recent study analyzed bacterial profiles from duodenal fluid in patients with pancreatic cysts, PDAC and controls as possible indicators of PDAC risk.^30^ PDAC patients displayed significantly decreased alpha-diversity and enrichment of *Fusobacterium*, *Enterococcus*, and *Bifidobacterium*, which, however, did not reach significance after multiple testing.^30^ Considering these findings, future comprehensive analyses of patient cohorts at risk for PDAC development may help to identify pro-tumorigenic microbiota signatures with predictive capacity.

The understanding of mechanisms by which abundant microbiota members like *Bacteroides* or *Parabacteroides* are involved in PDAC development is still limited. Potential modes of action might be inferred from data obtained in other tumor entities. Of interest, in multiple intestinal neoplasia (Min) mice (a mouse model for intestinal tumorigenesis) colonized with human enterotoxigenic *Bacteroides* fragilis the secreted toxin induced selective colonic signal transducer and activator of transcription-3 (Stat3) activation and colonic tumor formation.^31^^,^^32^ The effect was reversed by Interleukin-17 (IL) or IL-23 receptor blockade using specific antibodies. Enhanced Stat3 activation has been reported as important pro-tumorigenic signaling pathway in PDAC, operative also in KPC mice.^33^ However, *Bacteroides fragilis* has not been reported to be enriched in any human study reported thus far, nor was it observed in the models analyzed here.

Contrary to the observed enrichment of *P. distasonis* in the cancer models analyzed here, studies in a mouse model of azoxymethane (AOM)-induced colon cancer demonstrated a protective role of *P. distasonis* based on observed effects on stabilization of the intestinal epithelial barrier.^34^ Furthermore, in AOM-treated mice under a high-fat diet *P. distasonis* attenuated toll-like receptor 4 signaling and Akt (protein Kinase B) activation and thereby blocked colonic tumor formation.^35^ The enrichment of *Parabacteroides* observed here during PDAC development may indicate the relevance of mechanisms different from those in colon cancer. However, *Parabacteroides* was also enriched in Asian patients with early hepatocellular carcinoma. This data suggest that different mechanisms might be functional in various cancers depending on the cell type- and microenvironmental context.^36^

Of interest, increased Enterobacteriaceae and *Parabacteroides* relative abundances have been observed in two distinct mouse models with tumor cachexia.^37^ The authors concluded that these changes occur independently from food intake and are because of effects of cancer development. Noteworthy, frailty and chronic inflammatory states in humans have been correlated with increased levels of *Parabacteroides* and *Alistipes,* in individuals in long-term care.^38^ All these findings imply that the *Parabacteroides* relative abundance correlates with tumor development accompanied or even sustained by changes of inflammatory processes. Nevertheless, the specific influence and way of action of *Bacteroides* or *Parabacteroides* in PDAC development remains to be elucidated.

In addition to endocrine and exocrine function the pancreas secretes antimicrobial peptides. Deletion of the acinar Ca^2+^ channel Orai in mice led to high levels of mortality by bacterial outgrowth, dysbiosis and finally systemic translocation.^39^ Such effects may influence microbiome composition in animals over time and therefore could have been responsible for results depicted in our study. As we investigated early stages of pancreatic cancer development where most pancreatic acinar cells are not affected and large numbers of KC and KPC mice, we assume such effects not to be relevant for the findings of our study.^16^

In conclusion, our study in two different murine pancreatic cancer models showed distinct genotype, age, and gender specific alterations of the microbiome during tumor development. The use of two distinct tumor progression models confirmed that these alterations develop continuously with age and supports the possibility of a functionally relevant crosstalk between intestinal microbiome and genetically induced pancreatic lesions even at preinvasive, early stages.

Genera and species level data of our analysis as such identify suitable candidates like species of the *Bacteroides* genus or *P. distasonis* that should be further evaluated in their capacity to contribute to PDAC development.

The corroboration of distinct microbiota signatures associated with preneoplasia, and early tumorigenesis offers the opportunity to further develop diagnostic markers in humans and novel treatment targets. Nevertheless, more studies are needed to clarify mechanisms relevant in pancreatic carcinogenesis with a special focus on gender-related effects reported here.

### Limitations of the study

Our study is limited by the fact that no specific mechanistic insights in processes induced by genera specifically enriched or depleted in cancer models can be provided. Next, antimicrobial peptides secreted by the pancreas might corroborate results. In addition, the correlations observed in fecal samples have not been investigated for their spatial relevance. As such, it remains unclear which of the enriched or depleted genera are relevant in the pancreatic parenchyma or mediate their effects indirectly. However, the patterns retrieved are significant and consistent between the two most widely used murine PDAC models, supporting an important association between fecal microbiome composition and PDAC development.

## STAR★Methods

### Key resources table

**Table:** 

REAGENT or RESOURCES	SOURCE	IDENTIFIER
**Critical commercial assays**		

FastDNA Spin Kit for Soil	MP Biomedicals™	https://www.mpbio.com

**Deposited data**		

Sequencing datasets	NCBI SRA	PRJNA820878

**Experimental models: Organisms/strains**		

Pdx1-Cre	The Jackson Laboratory	RRID:IMSR_JAX:014647
LSL-Kras^G12D^	The Jackson Laboratory	RRID:IMSR_JAX:008179
LSL-p53^R172H^	The Jackson Laboratory	RRID:IMSR_JAX:008652

**Software and algorithms**		

MOTHUR	Patrick Schloss	https://mothur.org
SILVA	SILVA rRNA database project	https://www.arb-silva.de
RDP	Ribosomal Database Project	http://rdp.cme.msu.edu
Prism 7	Graphpad Software	https://www.graphpad.com
PRIMER v.7.0.11	PRIMER-E, Plymouth Marine Laboratory	https://Primer-e.com
JMP15	SAS Institute GmbH	https://www.sas.com

### Resource availability

#### Lead contact

Further information and requests for resources and reagents should be directed to and will be fulfilled by the lead contact Dietmar H. Pieper (dpi@helmholtz-hzi.de)

#### Materials availability

This study did not generate new unique reagents.

### Method details

#### Experimental model and subject details

In this study we used the spontaneous pancreatic carcinoma models KC (Kras^G12D/+^ expressing mice) and KPC (Kras^G12D/+^ p53^R172H/+^ expressing mice), that were generated by mating floxed LSL-Kras^G12D/+^ LSL-p53^R172H/+^ with homozygous Pdx1-Cre mice.^16^^,^^17^ Mice were housed under specific-pathogen-free conditions and fed with standard mouse chow. Littermates were genotyped after 2 and separated after 3–4 weeks with 1–4 animals living in each cage. Genotype specific housing prevented microbial contamination from one to the other genotype. Fecal samples were collected after 5, 11 and 17 weeks. All samples were collected from individual animals under pathogen-free conditions between 9 and 10 a.m. to avoid influence of circadian rhythm. At 17 weeks or when defined stopping criteria were given (KPC mice, n = 7, lifetime ≤105d/15 weeks), the animals were killed with cervical dislocation. KPC mice sacrificed before week 17 were analyzed within the overall KPC cohort. Tissue samples were flash frozen or formalin-fixed for paraffin embedding. Fecal specimens were stored in sterile tubes at −80°C until further use. The experimental design is summarized in Figure S6 and a detailed histological description as well as the gender distribution of the cohorts is described in Table S4. All animal experiments were conducted in accordance with the file reference MLU 2-1348 (Landesverwaltungsamt Sachsen-Anhalt).

#### DNA extraction and 16S rRNA gene amplicon sequencing

DNA was extracted using the FastDNA Spin Kit for Soil (MP Biomedicals^TM^) following the manufacturer’s instructions. A 2-step PCR-approach was used to amplify the V1-V2 variable region of the 16S rRNA gene. PCR with primers 27Fbif and 338R containing part of the sequencing primer sites as short overhangs (given in italics) (*ACGACGCTCTTCCGATCT*AGRGTTHGATYMTGGCTCAG and *GACGTGTGCTCTTCCGATCT*TGCTGCCTCCCGTAGGAGT, respectively) was used to enrich for target sequences (20 cycles). A second amplification step of 10 cycles added the two indices and Illumina adapters to amplicons.^40^ Obtained products were pooled in equimolar ratios and sequenced on an Illumina MiSeq (2X300 bases, San Diego, USA). Demultiplexed raw data for all the amplicon sequencing pair-end datasets are publicly available at the NCBI Sequence Reads Archive (SRA) under BioProject accession number PRJNA820878.

### Quantification and statistical analysis

Bioinformatic processing was performed as previously described.^41^ Raw reads were merged with the RDP assembler.^42^ Sequences were aligned within MOTHUR (gotoh algorithm using the SILVA reference database) and subjected to pre-clustering (diffs = 2) yielding so-called phylotypes that were filtered for an average abundance of ≥0.001% and a sequence length ≥250bp before analysis.^43^ Overall, 8.796.506 paired-ends reads were obtained with a mean of 46.055 ± 6951 reads per sample. All samples were re-sampled to equal the smallest library size of 25.366 reads using the phyloseq package returning 1560 phylotypes.^44^ Phylotypes were assigned to a taxonomic affiliation based on the naïve Bayesian classification with a pseudo-bootstrap threshold of 80%.^45^ Phylotypes were then manually analyzed against the RDP database using the Seqmatch function as well as against the NCBI database to define the discriminatory power of each sequence read. A species name was assigned to a phylotype when 16S rRNA gene fragments of previously described isolates of that species showed ≤2 mismatches with the respective representative sequence read.^41^ Relative abundances (in percentage) of phylotypes or genera were used for downstream analyses. Calculation of diversity indices (species richness S, Shannon diversity index H, Pielous evenness J, Simpson diversity index 1-ʌ and multivariate analyses were performed using PRIMER (v.7.0.11, PRIMER-E, Plymouth Marine Laboratory, UK), whereas univariate analyses were performed using Prism 7 (Graphpad Software, Inc.). Differences in diversity indices were tested for by ordinary ANOVA and 2-way ANOVA analysis where multiple comparisons were corrected using the Holm-Sidak test (comparisons of genotypes, J) or the Welch ANOVA test where multiple comparisons were corrected using the Dunnett T3 test (comparisons of genotypes, S, H and 1-ʌ), by the repeated measures ANOVA where multiple comparisons were corrected using the Tukey test (comparison of different ages, J) or the repeated measures ANOVA with the Geissler-Greenhouse correction where multiple comparisons were corrected using the Tukey test (comparison of different ages, S, H and 1-ʌ), or by unpaired t-test (comparison of different gender, J) or by the unpaired t-test with Welch's correction (comparison of different gender, S, H and 1-ʌ). Three-way ANOVA analysis taking into consideration the factors genotype, age and gender was performed using JMP15 (SAS Institute GmbH Heidelberg).

The data matrices comprising 1560 phylotypes, 87 genera or other taxa were used to construct sample-similarity matrices applying the Bray-Curtis algorithm, where samples were ordinated using non-metric multidimensional scaling (nMDS) with 50 random restarts.^46^^,^^47^ Significant differences between *a priori* predefined groups of samples were evaluated using Permutational Multivariate Analysis of Variance (PERMANOVA), allowing for type III (partial) sums of squares, fixed effects sum to zero for mixed terms. Monte Carlo p-values were generated using unrestricted permutation of raw data.^48^ Groups of samples were considered significantly different if the p-value was <0.05. The abundances of phyla, genera and of those phylotypes that were present in the community of at least 20% of the samples, were compared by the Kruskal-Wallis test with Benjamini-Hochberg corrections for multiple comparisons.^49^ Groups of samples were considered significantly different if the adjusted p-value was <0.05. Three-way ANOVA analysis was performed using JMP15 (SAS Institute GmbH Heidelberg) on square root transformed data. Groups of samples were considered significantly different if the Benjamini-Hochberg corrected p-value was <0.05. If multiple pairwise comparisons were performed values were corrected using the Tukey test.

## Data Availability

Demultiplexed raw data for all the amplicon sequencing pair-end datasets have been deposited at the NCBI Sequence Reads Archive (SRA) and are publicly available as of the date of the publication. Accession numbers are listed in the key resources table. This paper does not report original code. Any additional information required to reanalyze the data reported in this paper is available from the lead contact upon request or in the Supplementary Tables.

## Supplemental information

Document S1. Figures S1–S6 and Tables S3, S4, S5, S7, S8, S9, S10, and S15

Table S1. Nucleotide sequences of all phylotypes determined using Illumina-based amplicon deep-sequencing, their phylogenetic annotation and sequence count data across all 191 fecal samples, related to all Figures

Table S2. Relative abundance data of species level taxa, genera, families, orders, classes and phyla across all 191 fecal samples, related to all Figures

Table S6. Diversity indices across 191 samples, related to Figure 2

Table S11. One-way analysis of genera, families, orders, classes and phyla differentially distributed depending on age as well as their median and mean relative abundance, related to Figure 3, 4, 5 and 6

Table S12. One-way analysis of genera, families, orders, classes and phyla differentially distributed depending on genotype as well as their median and mean relative abundance, related to Figure 3, 4, 5 and 6

Table S13. One-way analysis of genera, families, orders, classes and phyla differentially distributed depending on gender as well as their median and mean relative abundance, related to Figure 3, 4, 5 and 6

Table S14. Three-way analysis of species level taxa differentially distributed depending on age, genotype and gender, the interactions between factors as well as the mean relative abundances of species level taxa, related to Figure 7
