# Supplementary material for: Gender-specific changes of the gut microbiome correlate with tumor development in murine models of pancreatic cancer
Source: iScience. 2023 May 18;26(6):106841. doi: 10.1016/j.isci.2023.106841 (PMC10225934; doi:10.1016/j.isci.2023.106841)
Supplement: Document S1. Figures S1–S6 and Tables S3, S4, S5, S7, S8, S9, S10, and S15 [file mmc1.pdf]

iScience, Volume *26*

Supplemental information

Gender-specific changes of the gut microbiome correlate with tumor development in murine models of pancreatic cancer

Tom Kaune, Heidi Griesmann, Katharina Theuerkorn, Monika Hämmerle, Helmut Laumen, Sebastian Krug, Iris Plumeier, Silke Kahl, Howard Junca, Luiz Gustavo dos Anjos Borges, Patrick Michl, Dietmar H. Pieper, and Jonas Rosendahl

Supplemental information

Table S 3. Factors influencing global community structures as indicated by PERMANOVAs main test, related to Figure 1

	Phylotype		Genus		Family		Order		Class		Phylum	
Factor	pseudo-F	p(MC)	pseudo-F	p(MC)	pseudo-F	p(MC)	pseudo-F	p(MC)	pseudo-F	p(MC)	pseudo-F	p(MC)
Age	4.53	**0.001**	5.53	**0.001**	6.22	**0.001**	7.10	**0.001**	7.14	**0.001**	13.95	**0.001**
Genotype	6.52	**0.001**	5.18	**0.001**	4.72	**0.001**	1.79	0.109	1.80	0.09	0.41	0.768
Gender	6.26	**0.001**	6.89	**0.001**	6.49	**0.001**	2.70	**0.048**	2.58	0.055	1.54	0.187
Age x gentoype	1.29	0.086	1.73	0.015	2.05	**0.023**	2.12	**0.017**	2.14	**0.027**	1.31	0.251
Age x gender	1.02	0.428	0.94	0.507	0.59	0.808	0.53	0.77	0.50	0.824	0.38	0.817
Genotype x gender	2.73	**0.002**	1.94	**0.037**	1.97	0.052	1.10	0.343	1.12	0.326	0.50	0.696
Age x genotype x gender	0.77	0.882	0.92	0.56	0.99	0.46	1.09	0.372	1.08	0.371	0.80	0.591

The influence of age, genotype and gender and their interaction on the microbiota composition was calculated by a three-way PERMANOVA (main test). The pseudo-F statistic and the Monte Carlo p-values are given for each comparison performed at different taxonomic levels (from phylotype to phylum).

Table S 4. Gender distribution and histological characteristics of the investigated cohorts, related to STAR Methods

**Tumor model**	**Remodelling [%]**	**ADM**		**AFL**		**PanIN**		**Tumor grading PDAC**				
		No	Yes	No	Yes	low grade	high grade	G0	G2	G2-3	G3	M
KC (n_{female} = 7 n_{male} = 13)	7.2 (mean)<br>5 (median)<br>1-37 (range)	2/20	17/20	17/20	2/20	18/20	0/20	19/20	0/20	0/20	0/20	0/20
KPC (n_{female} = 11 n_{male} = 17)	48.2 (mean)<br>45 (median)<br>5-100 (range)	2/28	26/28	13/28	15/28	23/28	3/28	17/28	8/28	6/28	3/28	6/28

In total 16 PDX1-Cre, 20 KC and 28 KPC mice were analyzed in this study. The histopathological analysis of the control mice did not reveal any relevant changes. Seven KPC (n_{female} = 2**,** n_{male} = 5) mice had to be sacrificed before they reached the age of 17 weeks (between 13 and 15 weeks). ADM, Acinar to ductal metaplasia; AFL, Atypic flat lesions; PanIN, Pancreatic intraepithelial neoplasia; Tumor grading of Pancreatic ductal adenocarcinoma (PDAC): G0, no tumor; G2-G3, moderately to poorly differentiated tumor; M, liver metastasis.

Table S 5. Factors influencing global community structures as indicated by PERMANOVAs pairwise tests, related to Figure 1

Factor	Groups	Phylotype		Genus		Family		Order		Class		Phylum	
		t	p(MC)	t	p(MC)	t	p(MC)	t	p(MC)	t	p(MC)	t	p(MC)
M	KC, KPC	2.02	**0.001**	1.63	**0.023**	1.35	0.119	0.96	0.397	0.94	0.467	0.62	0.675
	KC, Cre	2.13	**0.001**	1.58	**0.023**	1.16	0.231	0.91	0.466	0.91	0.466	0.48	0.827
	KPC, Cre	2.09	**0.001**	1.74	**0.011**	1.48	0.061	0.92	0.452	0.92	0.446	0.92	0.402
F	KC, KPC	1.41	**0.031**	1.21	0.185	1.22	0.192	0.79	0.568	0.78	0.591	0.84	0.435
	KC, Cre	2.51	**0.001**	2.42	**0.001**	2.58	**0.001**	1.86	**0.031**	1.88	**0.022**	0.62	0.638
	KPC, Cre	2.70	**0.001**	2.57	**0.003**	2.65	**0.002**	1.60	0.09	1.61	0.064	0.35	0.881
KC	M, F	2.10	**0.001**	2.27	**0.001**	2.18	**0.003**	1.16	0.224	1.17	0.254	1.12	0.274
KPC	M, F	2.30	**0.001**	2.15	**0.003**	2.07	**0.004**	0.85	0.526	0.83	0.536	0.99	0.359
Cre	M, F	1.71	**0.003**	1.50	0.063	1.63	0.050	1.64	0.060	1.62	0.057	0.66	0.664
5 weeks	KC, KPC	0.98	0.459	1.07	0.301	1.09	0.311	0.98	0.377	0.99	0.400	1.63	0.097
	KC, Cre	1.46	**0.028**	1.63	**0.036**	1.69	0.054	1.56	0.073	1.57	0.086	0.75	0.510
	KPC, Cre	1.84	**0.004**	2.12	**0.010**	2.21	**0.013**	1.97	**0.030**	1.97	**0.031**	0.86	0.419
11 weeks	KC, KPC	1.09	0.290	1.30	0.121	1.24	0.196	1.31	0.163	1.31	0.173	1.48	0.108
	KC, Cre	2.05	**0.001**	2.03	**0.003**	2.07	**0.007**	1.83	**0.033**	1.83	**0.016**	0.96	0.393
	KPC, Cre	2.03	**0.001**	1.70	**0.018**	1.71	**0.030**	1.20	0.237	1.19	0.220	0.90	0.442
17 weeks	KC, KPC	1.56	**0.007**	1.57	**0.025**	1.58	**0.048**	0.97	0.404	0.97	0.440	0.17	0.968
	KC, Cre	2.06	**0.001**	1.67	**0.008**	1.61	**0.038**	1.45	0.114	1.46	0.112	0.58	0.728
	KPC, Cre	2.31	**0.001**	2.01	**0.004**	2.02	**0.003**	1.24	0.192	1.26	0.199	0.73	0.569
KC	1, 2	1.22	0.140	1.22	0.184	1.27	0.166	1.31	0.175	1.31	0.158	1.51	0.119
	1, 3	1.68	**0.005**	1.68	**0.014**	1.82	**0.025**	2.04	**0.013**	2.04	**0.015**	1.89	**0.041**
	2, 3	0.95	0.519	0.90	0.518	0.84	0.568	0.86	0.550	0.86	0.548	0.75	0.557
KPC	1, 2	2.06	**0.002**	2.59	**0.002**	2.74	**0.003**	3.11	**0.001**	3.12	**0.002**	4.70	**0.001**
	1, 3	2.31	**0.001**	2.46	**0.001**	2.52	**0.001**	2.78	**0.001**	2.79	**0.002**	3.65	**0.001**
	2, 3	1.48	**0.021**	1.76	**0.010**	1.81	**0.010**	0.78	0.624	0.82	0.571	1.22	0.190
Cre	1, 2	1.34	0.064	1.48	**0.042**	1.65	0.054	1.73	0.055	1.73	**0.028**	2.63	**0.001**
	1, 3	1.60	**0.011**	1.66	**0.041**	1.77	**0.048**	1.88	**0.036**	1.88	**0.033**	1.95	**0.032**
	2, 3	1.38	**0.042**	1.86	**0.009**	2.19	**0.004**	2.21	**0.009**	2.22	**0.009**	0.58	0.756

The interaction in the effects of genotype and gender as well as those of genotype and age on the variability of microbial assemblages was calculated by PERMANOVA (interaction between factors). The t statistics and the Monte Carlo p-values are given for paired tests among levels of the factors gender (M=male, F=female), genotype (Cre-PDX1, KC, KPC) and age (1, 5 weeks; 2, 11 weeks; 3, 17 weeks of age). Analysis was performed at different taxonomic levels (from phylotype to phylum).

Table S 7. Richness and diversity of fecal microbial communities as assessed by one factor analyses, related to Figure 2

Groups	No. of phylotypes S		p	Shannon index H		p	Pielous evenness J		p	Simpsons index (1-ʌ)		p
	Group I	Group II		Group I	Group II		Group I	Group II		Group I	Group II	
Cre, KC	562 ± 49	544 ± 59	0.2325	4.245 ± 0.304	4.293 ± 0.277	0.7807	0.6705 ± 0.0427	0.6819 ± 0.0383	0.3882	0.9665 ± 0.0172	0.973 ± 0.0125	0.09
Cre, KPC	562 ± 49	533 ± 83	**0.0391**	4.245 ± 0.304	4.186 ± 0.392	0.7124	0.6705 ± 0.0427	0.6672± 0.0497	0.9700	0.9665 ± 0.0172	0.965 ± 0.0226	0.9654
KC, KPC	544 ± 59	533 ± 83	0.7315	4.293 ± 0.277	4.186 ± 0.392	0.1641	0.6819 ± 0.0383	0.6672± 0.0497	0.1371	0.9730 ± 0.0125	0.965 ± 0.0226	**0.0241**
1, 2	529 ± 60	549 ± 63	0.0720	4.199 ± 0.333	4.219 ± 0.321	0.9149	0.6698 ± 0.046	0.6692 ± 0.0432	0.9950	0.9645 ±0.219	0.9679 ± 0.154	0.3547
1, 3	529 ± 60	554 ± 82	**0.0360**	4.199 ± 0.333	4.279 ± 0.360	0.3077	0.6698 ± 0.046	0.6781 ± 0.0449	0.4661	0.9645 ±0.219	0.9709 ± 0.185	0.0911
2, 3	549 ± 63	554 ± 82	0.8912	4.219 ± 0.321	4.279 ± 0.360	0.4599	0.6692 ± 0.0432	0.6781 ± 0.0449	0.4104	0.9679 ± 0.154	0.9709 ± 0.185	0.3547
M, F	536 ± 77	557 ± 52	**0.0224**	4.192 ± 0.366	4.301 ± 0.280	**0.0207**	0.6676 ± 0.0475	0.6805 ± 0.0395	0.0523	0.9580 ± 0.021	0.9711 ± 0.014	**0.0363**

Diversity was assessed by total phylotype number, Shannon diversity (H'), Pielou's evenness (J') and Simpsons diversity (1-ʌ), respectively with genotype (Cre-PDX1, KC and KPC), age (1=5 weeks, 2=11 weeks and 3=17 weeks) and gender (F=female, M=male) as factors. The mean ± sd (standard deviation) is given. P-values were calculated by ANOVA where multiple comparisons were corrected using the Holm-Sidak test (comparisons of genotypes, J) or the Welch ANOVA test where multiple comparisons were corrected using the Dunnett T3 test (comparisons of genotypes, S, H and 1-ʌ), by the repeated measures ANOVA where multiple comparisons were corrected using the Tukey test (comparison of different ages, J) or the repeated measures ANOVA with the Geissler-Greenhouse correction where multiple comparisons were corrected using the Tukey test (comparison of different ages, S, H and 1-ʌ), or by unpaired t-test (comparison of different gender, J) or by the unpaired t-test with Welch´s correction (comparison of different gender, S, H and 1-ʌ).

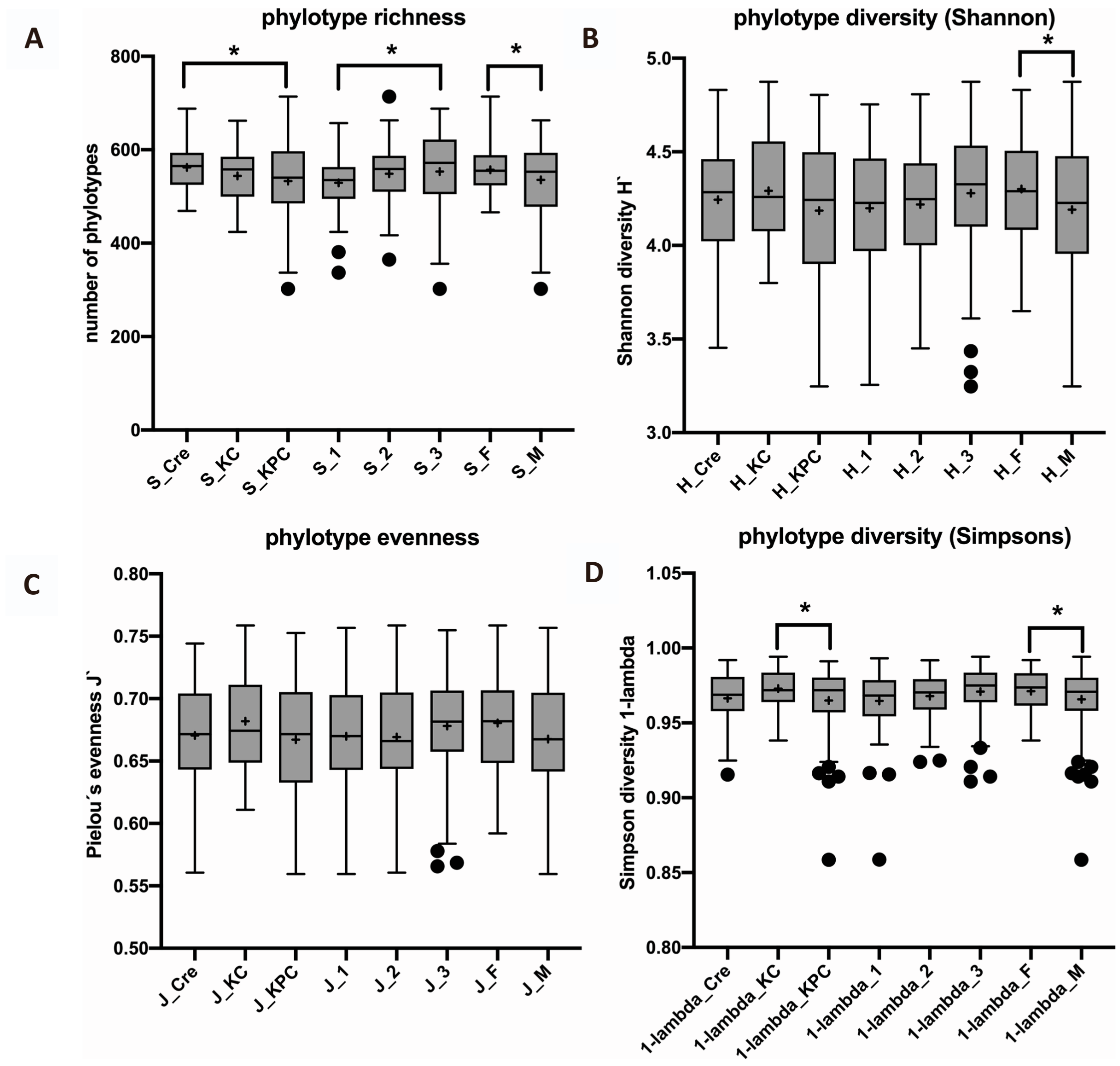

Figure S 1: Fecal bacterial community diversity of the overall cohorts, related to Figure 2. Diversity is indicated by (A) total phylotype number, (B) Shannon diversity (H’), (C) Pielou’s evenness (J’) and (D) Simpsons diversity (1-λ), respectively. Statistically significant differences of distinct genotypes (Cre-PDX1, KC and KPC), age (1=5 weeks, 2=11 weeks and 3=17 weeks) or between gender (F=female, M=male) are indicated by *$p<0.05$. The mean is indicated by + and the median by a black line. The box represents the interquartile range. The whiskers extend to the upper adjacent value (largest value = 75th percentile + 1.5 x IQR) and the lower adjacent value (lowest value = 25th percentile - 1.5 x IQR) and dots represent outliers.

Table S 8. Richness and diversity of fecal microbial communities as assessed by two factor analyses using genotype and age as factors, related to Figure 2.

Factor	Groups compared	No. of phylotypes S		p	Shannon index H		p	Pielous evenness J		p	Simpsons index (1-λ)		p
	Group I/Group II	Group I	Group II		Group I	Group II		Group I	Group II		Group I	Group II	
Cre	1, 2	533.6	558.4	0.2208	0.6526	0.6621	0.7610	4.097	4.188	0.7510	0.9565	0.9663	0.2899
	1, 3	533.6	594.3	**0.0081**	0.6526	0.6967	**0.0040**	4.097	4.449	**0.0156**	0.9565	0.9767	**0.0088**
	2, 3	558.4	594.3	0.0853	0.6621	0.6967	**0.0307**	4.188	4.449	**0.0122**	0.9663	0.9767	0.0594
KC	1, 2	530.8	546.8	0.2427	0.6789	0.6822	0.9596	4.257	4.299	0.8311	0.9711	0.9732	0.8519
	1, 3	530.8	555.0	0.2017	0.6789	0.6844	0.8923	4.257	4.322	0.6623	0.9711	0.9745	0.6634
	2, 3	546.8	555.0	0.7092	0.6822	0.6844	0.9820	4.299	4.322	0.9405	0.9732	0.9745	0.8764
KPC	1, 2	525.1	544.3	0.5234	0.6733	0.6636	0.6233	4.215	4.178	0.8769	0.9644	0.9648	0.9945
	1, 3	525.1	528.1	0.9807	0.6733	0.6624	0.5453	4.215	4.148	0.7020	0.9644	0.9647	0.9967
	2/3	544.3	528.1	0.7167	0.6636	0.6624	0.9916	4.178	4.148	0.9418	0.9648	0.9647	0.9997
5 weeks	Cre, KC	533.6	530.8	0.9842	0.6526	0.6789	0.1771	4.097	4.257	0.2413	0.9565	0.9711	**0.0299**
	Cre, KPC	533.6	525.1	0.8784	0.6526	0.6733	0.2982	4.097	4.215	0.4960	0.9565	0.9644	0.4878
	KC, KPC	530.8	525.1	0.9499	0.6789	0.6733	0.8999	4.257	4.215	0.9019	0.9711	0.9644	0.5167
11 weeks	Cre, KC	558.4	546.8	0.6890	0.6621	0.6822	0.3611	4.188	4.299	0.4677	0.9663	0.9732	0.2704
	Cre, KPC	558.4	544.3	0.7248	0.6621	0.6636	0.9933	4.188	4.178	0.9945	0.9663	0.9648	0.9546
	KC, KPC	546.8	544.3	0.9915	0.6822	0.6636	0.3255	4.299	4.178	0.4041	0.9732	0.9648	0.1326
17 weeks	Cre, KC	594.3	555.0	0.1257	0.6967	0.6844	0.6843	4.449	4.322	0.3125	0.9767	0.9745	0.8785
	Cre, KPC	594.3	528.1	**0.0131**	0.6967	0.6624	**0.0377**	4.449	4.148	**0.0123**	0.9767	0.9647	0.0960
	KC, KPC	555.0	528.1	0.5164	0.6844	0.6624	0.2072	4.322	4.148	0.2262	0.9745	0.9647	0.1584

Diversity was assessed by total phylotype number, Shannon diversity (H'), Pielou's evenness (J') and Simpsons diversity (1-λ), respectively with genotype (Cre-PDX1, KC and KPC), and age (1=5 weeks, 2=11 weeks and 3=17 weeks) as factors. The mean is given. P-values were calculated by 2-way ANOVA where multiple comparisons were corrected using the Holm-Sidak test (comparisons of genotypes, J) or the Welch ANOVA test where multiple comparisons were corrected using the Dunnett T3 test (comparisons of genotypes, S, H and 1-ʌ), by the repeated measures ANOVA where multiple comparisons were corrected using the Tukey test (comparison of different ages, J) or the repeated measures ANOVA with the Geissler-Greenhouse correction where multiple comparisons were corrected using the Tukey test (comparison of different ages, S, H and 1-ʌ).

Table S 9. Richness and diversity of fecal microbial communities as assessed by two factor analyses using genotype and gender as factors related to Figure 2.

Factor	Groups compared	No. of phylotypes S		p	Shannon index H		p	Pielous evenness J		p	Simpsons index (1-λ)		p
	Group I, Group II	Group I	Group II		Group I	Group II		Group I	Group II		Group I	Group II	
F	Cre, KC	561.0	560.3	>0.9999	0.6720	0.6761	0.9859	4.254	4.277	0.9942	0.9685	0.9703	0.9850
	Cre, KPC	561.0	552.4	0.9582	0.6720	0.6890	0.4184	4.254	4.348	0.6704	0.9685	0.9734	0.7078
	KC, KPC	560.3	552.4	0.9668	0.6761	0.6890	0.6468	4.277	4.348	0.8267	0.9703	0.9734	0.9009
M	Cre, KC	563.0	535.5	0.2839	0.6693	0.6849	0.3875	4.237	4.301	0.8256	0.9649	0.9744	0.1084
	Cre, KPC	563.0	518.8	**0.0202**	0.6693	0.6535	0.3386	4.237	4.084	0.1479	0.9649	0.9597	0.5482
	KC, KPC	535.5	518.8	0.5782	0.6849	0.6535	**0.0024**	4.301	4.084	**0.0068**	0.9744	0.9597	**0.0006**
Ce	F, M	561.0	563.0	0.9994	0.6720	0.6693	0.9949	4.254	4.237	0.9974	0.9685	0.9649	0.8680
KC	F, M	560.3	535.5	0.4414	0.6761	0.6849	0.8384	4.277	4.301	0.9907	0.9703	0.9744	0.7877
KPC	F, M	552.4	518.8	0.0854	0.6890	0.6535	**0.0011**	4.348	4.084	**0.0014**	0.9734	0.9597	**0.0029**

Diversity was assessed by total phylotype number, Shannon diversity (H'), Pielou's evenness (J') and Simpsons diversity (1-λ), respectively with genotype (Cre-PDX1, KC and KPC) and gender (F=female, M=male) as factors. The mean is given and p-values were calculated by 2-way ANOVA where multiple comparisons were corrected using the Holm-Sidak test (comparisons of genotypes, J) or the Welch ANOVA test where multiple comparisons were corrected using the Dunnett T3 test (comparisons of genotypes, S, H and 1-ʌ), by the unpaired t-test (comparison of different gender, J) or by the unpaired t-test with Welch´s correction (comparison of different gender, S, H and 1-ʌ).

Table S 10. Richness and diversity of fecal microbial communities of Cre-PDX1 mice related to Figure 2.

Groups compared	No. of phylotypes S		p	Shannon index H		p	Pielous evenness J		p	Simpsons index (1-λ)		p
	Group I	Group II		Group I	Group II		Group I	Group II		Group I	Group II	
F1, F2	529.1	555	0.6303	0.6382	0.6723	0.364	4.003	4.248	0.3854	0.9552	0.9695	0.1944
F1, F3	529.1	598.7	0.0656	0.6382	0.7055	**0.0399**	4.003	4.511	**0.0367**	0.9552	0.9809	**0.0156**
F2, F3	555.0	598.7	0.2918	0.6723	0.7055	0.3812	4.248	4.511	0.3384	0.9695	0.9809	0.3412
M1, M2	537.0	561.1	0.4383	0.6638	0.6542	0.8447	4.171	4.141	0.9696	0.9575	0.9637	0.7118
M1, M3	537.0	590.9	**0.0319**	0.6638	0.6898	0.3145	4.171	4.400	0.1986	0.9575	0.9734	0.1394
M2, M3	561.1	590.9	0.2938	0.6542	0.6898	0.1297	4.141	4.400	0.1337	0.9637	0.9734	0.4556

Diversity was assessed by total phylotype number, Shannon diversity (H'), Pielou's evenness (J') and Simpsons diversity (1-λ) with age (1=5 weeks, 2=11 weeks and 3=17 weeks) and gender (F=female, M=male) as factors. The mean is given and p-values were calculated by repeated measures ANOVA where multiple comparisons were corrected using the Tukey test.

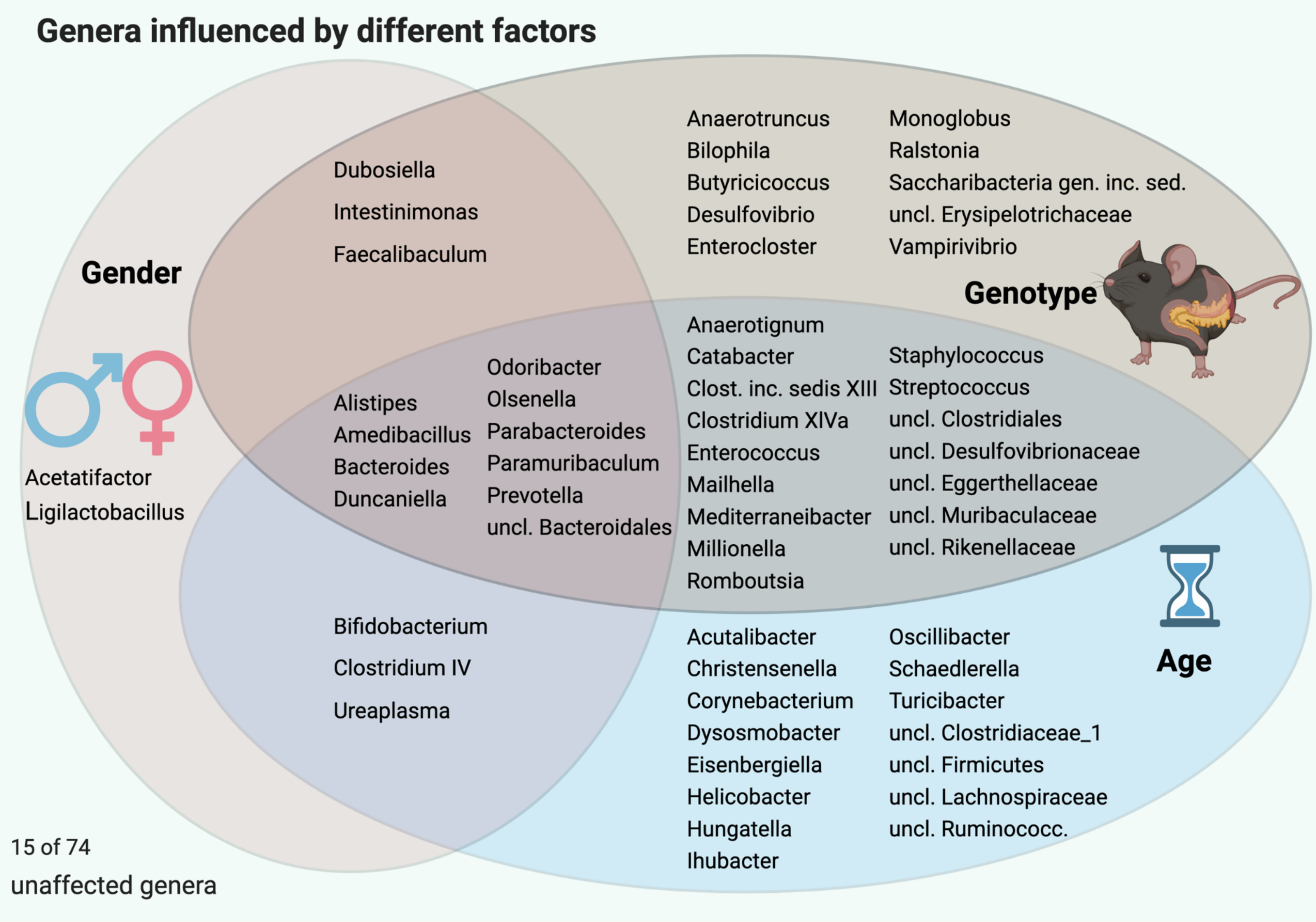

Figure S 2: Genera influenced by the factors gender genotype, and age or their combination, related to Figure 3. Overview of the 59 out of 74 genera affected by one or more of the factors (intersections). The figure was made in ©BioRender - biorender.com.

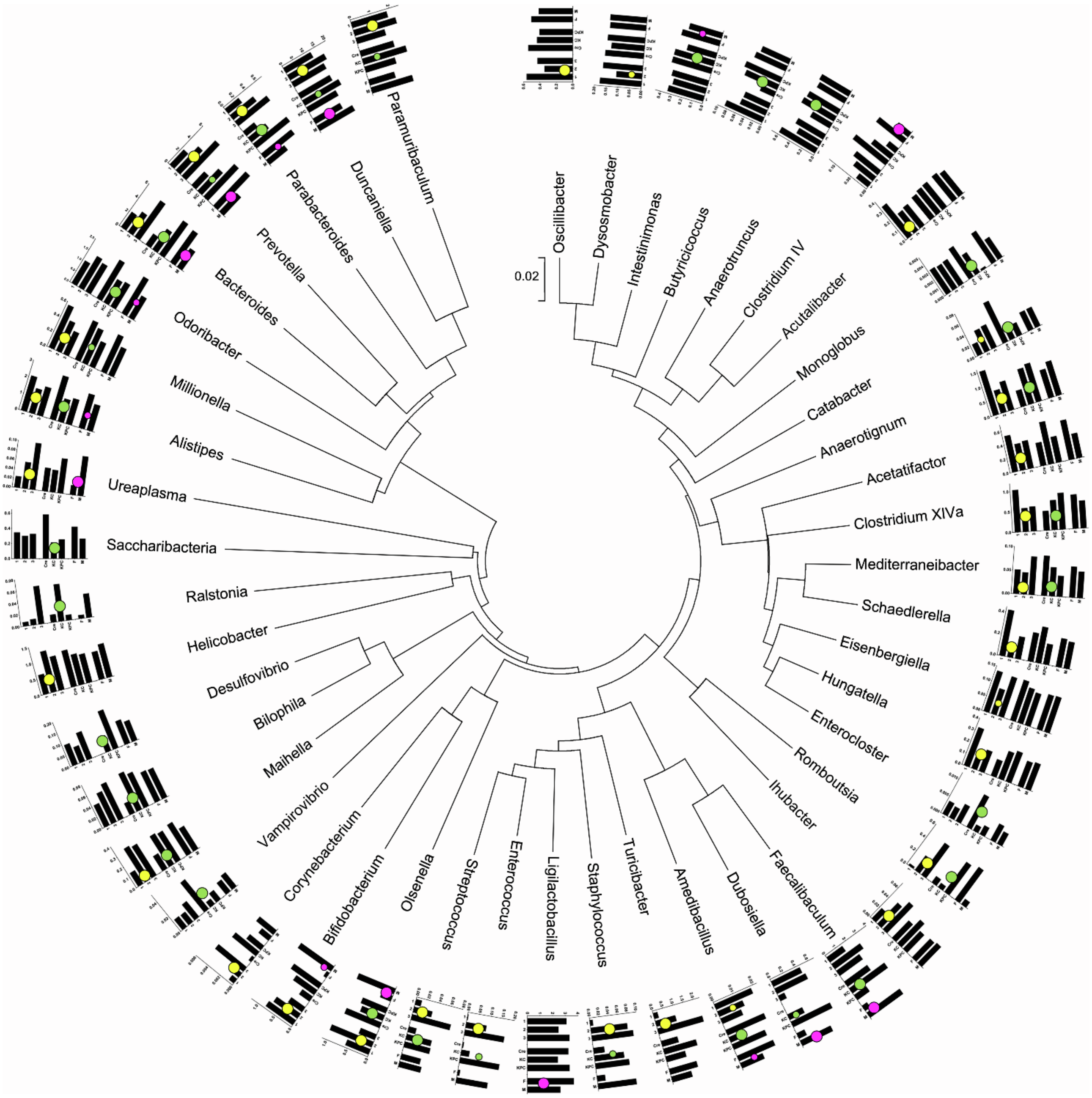

Figure S 3: Phylogenetic relationship among genera influenced by genotype, age and gender, related to Figure 3. The phylogenetic tree is based on representative full length 16S rRNA gene sequences (see Table S 7). The evolutionary history was inferred using the Neighbor-Joining method. The evolutionary distances were computed using the p-distance method and are in the units of the number of base substitutions per site. The relative mean abundances under different conditions are given as insert. A significant effect of genotype, age or gender on the relative abundance is indicated by green, yellow and magenta circles, with a large circle indicating a p-value <0.01 and a small circle indicating a p-value between 0.05 and 0.01.

Table S 15. Sequences used as representative for genera identified, related to Figure S4

No	Strain/sequence
1	Bifidobacterium adolescentis (T); ATCC 15703; AP009256
2	Corynebacterium mastitidis (T);S-8; CECT 4843; Y09806
3	Olsenella uli (T); ATCC49627; AF292373
4	Bacteroides fragilis (T); ATCC 25285 = NCTC 9343; CR626927
5	Parabacteroides distasonis (T); JCM 5825; AB238922
6	Prevotella copri (T); CB7; AB064923
7	Alistipes onderdonkii; JCM 16771; AB554231
8	uncultured rumen bacterium (Millionella); BRC141; EF436427
9	Porphyromonadaceae bacterium C941 (Duncaniella); JF803519
10	uncultured bacterium (Paramuribaculum); SWPT13_aaa04d04; EF097024
11	Odoribacter splanchnicus (T); NCTC 10825; L16496
12	Ralstonia solanacearum (T); LMG 2299; EF016361
13	Vampirovibrio chlorellavorus (T); ICPB 3707; HM038000
14	Bilophila wadsworthia (T); 7959; AJ867049
15	Desulfovibrio intestinalis (T); KMS2; Y12254
16	uncultured bacterium (Maihella); WD6_aak49b02; EU510775
17	Ureaplasma parvum (T); ATCC27815; AF073456
18	Candidatus Saccharibacteria bacterium GW2011_GWC2_44_17; CP011211
19	Staphylococcus saprophyticus (T); ATCC 15305; AP008934
20	Enterococcus faecalis (T); JCM 5803; AB012212
21	Ligilactobacillus salivarius (T); ATCC 11741; AF089108
22	Streptococcus dysgalactiae (T); ATCC 43078; AB002485
23	Eubacterium sp. WAL 18692 (Ihubacter); GQ461730
24	Clostridium aminophilum (Clostridium XIVa); 152R-1b; DQ278862
25	Eisenbergiella sp. AT9; LN881600
26	Clostridiales bacterium CIEAF 015 (Acetatifactor); AB702929
27	Hungatella hathewayi (T); DSM 13479 = CCUG 43506; AJ311620
28	Anaerotignum propionicum (T); JCM 1430; AB649276
29	Mediterraneibacter massiliensis (T); AT10; LN881607
30	Clostridium sp. ASF502 (Schaedlerella); ASF 502; AF157053
31	Enterocloster clostridioformis (T); ATCC 25537; M59089
32	Anaerotruncus colihominis (T); 14565; AJ315980
33	Butyricicoccus pullicaecorum (T); 25-3; EU410376
34	Oscillibacter ruminantium (T); GH1; JF750939
35	Clostridium leptum (T) (Clostridium IV); DSM 753T; AJ305238
36	Intestinimonas butyriciproducens (T); SRB-521-5-I; KC311367

37	Clostridium sp. Clone-25 (Acutalibacter); AB622839
38	uncultured bacterium (Monoglobus); E358; DQ326876
39	uncultured bacterium (Dysosmobacter); B5_253; EU766390
40	Catabacter hongkongensis (T); HKU16; AY574991
41	Turicibacter sanguinis; AF349724
42	Faecalibaculum rodentium (T); ALO17; KP881689
43	uncultured bacterium (Dubosiella); C21_m01; AY993273
44	Amedibacillus dolichus (T); ATCC 29143; L34682
45	Helicobacter hepaticus (T); Hh-2; U07574
46	Romboutsia lituseburensis (T); ATCC 25759; M59107

The strain or sequence name as well as accession number is given. Phylogenetic placement as performed by RDP is given in parentheses.

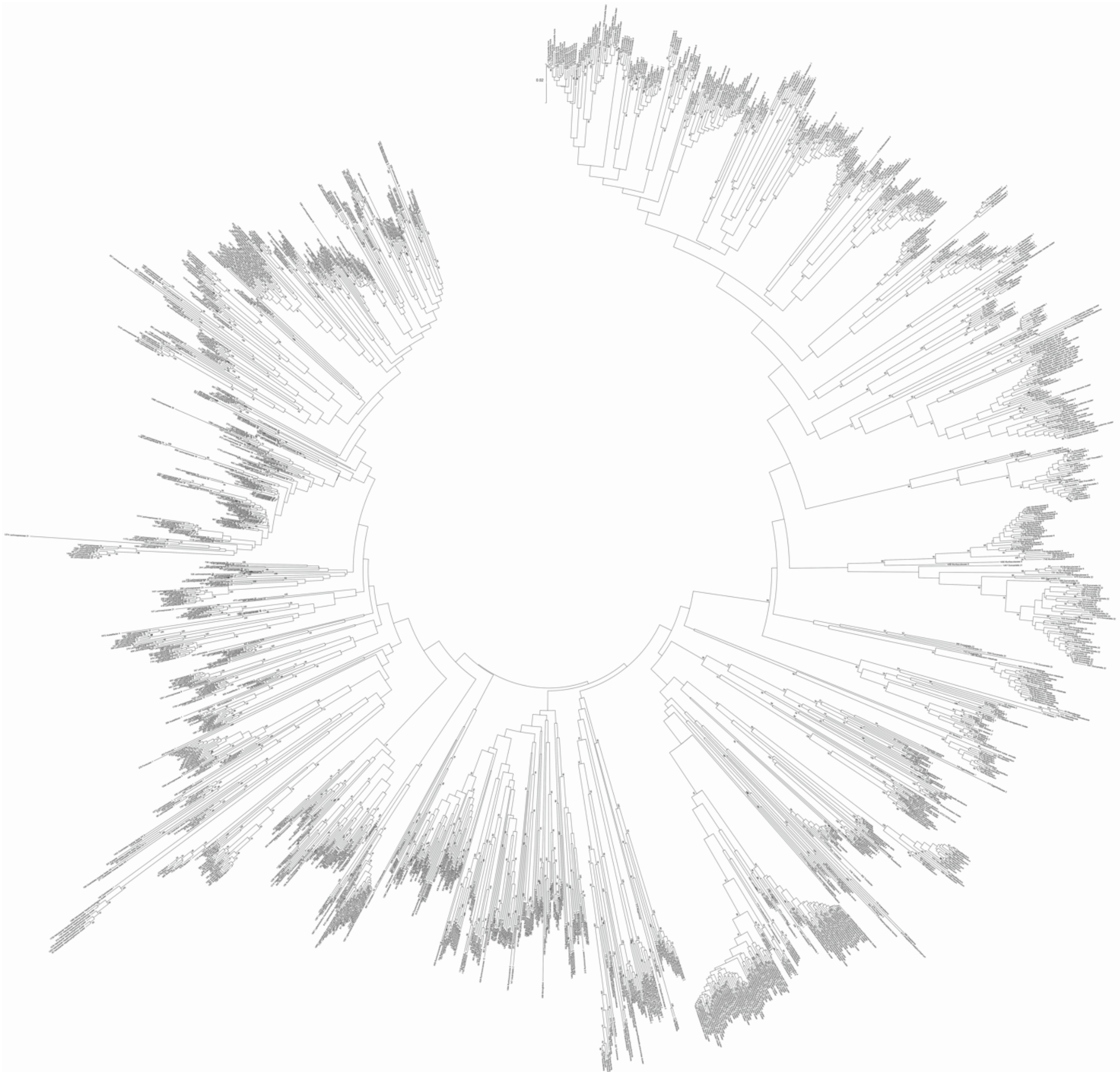

Figure S 4: Phylogenetic relationship among sequence clusters, related to Figure 7. The phylogenetic tree is based on partial 16S rRNA gene sequences. The evolutionary history was inferred using the Neighbor-Joining method. The evolutionary distances were computed using the p-distance method and are in the units of the number of base substitutions per site. Bootstrap values >50 are indicated.

Genus/species	Family	age	genotype	gender	genotype X gender	age X gender	genotype X age
Acetatifactor 1	Lachnospiraceae			F			
Anaerotignum 1		▼▼*	▲▲*				
Anaerotignum 4		*▲*	*▲*			*	
Anaerotignum 5		▲▲*	▼▼*				*
Clostridium XIVa 1		▼▾*	*▲*	F			*
Clostridium XIVa 2		▼**	▴▴*				
Hungatella 2		▼▾*					
Mediterraneibacter 3			▼▼*				
Mediterraneibacter 4		*▲▴					
Lachnospiraceae 1			▼▼*		**		
Lachnospiraceae 5		▼**	▼▼*		**		
Lachnospiraceae 9		▼▼*					
Lachnospiraceae 10		▲▲*					
Lachnospiraceae 11		*▲▴	▼▾*				
Lachnospiraceae 18		**▴			*		
Lachnospiraceae 20		▼▼*					
Lachnospiraceae 24			*▼▼				
Lachnospiraceae 26		*▲▴	*▼*				
Lachnospiraceae 27			▼▼*	F			***
Lachnospiraceae 28							
Lachnospiraceae 29					****		
Lachnospiraceae 30		▼▾*					
Lachnospiraceae 31		▼▼*					
Lachnospiraceae 32				F			
Lachnospiraceae 34					*		
Lachnospiraceae 37			▼▼*				**
Lachnospiraceae 38		▼▾*					
Lachnospiraceae 43			*▲*				
Lachnospiraceae 44			▲▲*		*		
Lachnospiraceae 45					****		
Lachnospiraceae 47			▼▼*				
Lachnospiraceae 48					**		
Lachnospiraceae 49					*		
Lachnospiraceae 52		▲▲*					**
Lachnospiraceae 57			▼▼*		*		
Lachnospiraceae 60		*▲*	▼**		**		
Lachnospiraceae 63		▼▼*					
Lachnospiraceae 64		▼▼*					
Lachnospiraceae 67				F			
Lachnospiraceae 70			▲*▾				
Lachnospiraceae 72		▲▴*					
Lachnospiraceae 74		▼*▲					
Lachnospiraceae 76					**		
Lachnospiraceae 77			*▲*				
Lachnospiraceae 81			▼▼*				
Flintibacter 2	Ruminococcaceae				*		
Intestinimonas 1							**
Intestinimonas 2		▲▲*	▼▼*				
Intestinimonas 4						*	
Oscillibacter 3		*▴▴	*▼*		*		***
Ruminococcaceae 1			▾▼*				
Ruminococcaceae 2			▼▼*		*		
Ruminococcaceae 4				M	*		
Ruminococcaceae 5		▼▼*					
Ruminococcaceae 8		▲▲*		M	**		
Ruminococcaceae 10		*▲▴					
Ruminococcaceae 11			▼**				
Ruminococcaceae 12			▴▲*				
Ruminococcaceae 15		▲▴*	▼▼*				
Ruminococcaceae 17					*		
Ruminococcaceae 18					*		
Ruminococcaceae 19			*▲*				

Genus/species	Family	age	genotype	gender	genotype X gender	age X gender	genotype X age
D. muris	Muribaculaceae	*▲*	▲▲*		*		
D. duboisii		*▼▾	*▼▼	M	****		
D. freteri		▲▲*	▲▲*				
Duncaniella 2			▲▼▾	M			
Duncaniella 4		▲▲*	▲▲*	F			
Duncaniella 5		▲▲*	▼▾*				
Duncaniella 6			▲▴▾				
Duncaniella 7			▴*▾	M			
Duncaniella 9		▲*▼	▼▼*	F		*	**
Duncaniella 13		▲▲*					
Duncaniella 16			▲*▾	M			
Duncaniella 17		▲▲*					
Duncaniella 18		▲▴*		F		*	
Duncaniella 19					*		
Duncaniella 22			*▼▼	M			
Muribaculum 1			▼▾▴				
M. intestinale		▲*▾					
Muribaculaceae 1		▲**					
Muribaculaceae 2			▾*▲				
Muribaculaceae 3		▾▼*	▴**				
Muribaculaceae 6		▲▲*	▲▲*	F			
Muribaculaceae 7		▲▲*	▼▼*	F		***	
Muribaculaceae 8		▴▲*	▾▼▾				
Muribaculaceae 9		▲**	*▼*		*		
Clostridiales IS XIII 1	Clostridiales IS XIII		▼▼*				*
Clostridiales 3					*		
Lactobacillus 3	Lactobacillaceae			F	*		
Ligilactobacillus 1				F	*		
Streptococcus 1	Streptococcaceae	▲▲*	▲▲*				
Erysipelotrichaceae 1	Erysipelotrichaceae		*▲▲				
Erysipelotrichaceae 2			*▲*				
Erysipelotrichaceae 4		▾**	▼**				
B. acidifaciens (A24 cluster	Bacteroidaceae	▲▲*	▼▾*				
B. acidifaciens (type cluster)		*▲▲	▲▲▲	F	*		*
B.uniformis		*▴*	*▲*	F			
Bacteroides 1		*▲*	▲▲*	F	*		
Bacteroides 2		*▲*	▲▲*				
Bacteroides 3		*▲*	▲▲*	F	**		
Bacteroides 4			▴**	F			
Bacteroides 5		*▲*	▲▲*	F	*	*	
Bacteroides 6		*▲*	▲▲*	F	*		
Alistipes 1	Rikenellaceae	▲▲*					
Alistipes 2		▲▲*		F	***		
P. diastonis	Porphyromonadaceae	▲▲*	▴▲▲	F	*		
Parabacteroides		▲**	*▲▴		*		
Prevotella 1	Prevotellaceae	▲▲*	▼▼*	F			
Prevotella 3		*▲*	▲▲*	F	****		
Odoribacter 1	Odoribacteraceae		▼▼*	F			
Odoribacter 2				F			
Odoribacter 3			▼▼▴				
Bacteroidales 1		▲▴*		F			
Bacteroidales 2			*▲▲	F			
Adlercreutzia mucosicola	Eggerthellaceae			F			
Eggerthellaceae 2			▼▼*				
Eggerthellaceae 3			▾**	F			
Eggerthellaceae 4			▼▼*				
Desulfovibrionaceae 1	Desulfovibrionaceae	▼▼*	▼▼▼				***

Figure S 5: **Species level taxa significantly influenced by genotype, age or gender, related to Figure 7.** Square root transformed relative abundance date dependent on age, genotype and gender were analyzed by three-way ANOVA. Factors influencing the relative abundance are indicated in yellow (age), green (genotype) or magenta (gender) if p<0.01 and by orange (age), light green (genotype) or light magenta (gender) if p=0.01-0.05. Significant interactions between factors are indicted in blue or light blue with *, p<0.05; **, p<0.01; ***, p<0.001; ****, p<0.0001. A significant increase (p<0.01) with age is indicated by a large ▲, a decrease is indicated by a large ▼. A significant increase/decrease with p=0.01-0.05 is indicated by the small symbols ▴ or ▾. The comparisons given are 5 weeks/11 weeks, 5 weeks/17 weeks and 11 weeks/17 weeks and Pdx1-Cre/KC, Pdx1-Cre/KPC and KC/KPC, respectively. In case of gender, the gender with the higher abundance is indicated as F (female) or M (male).

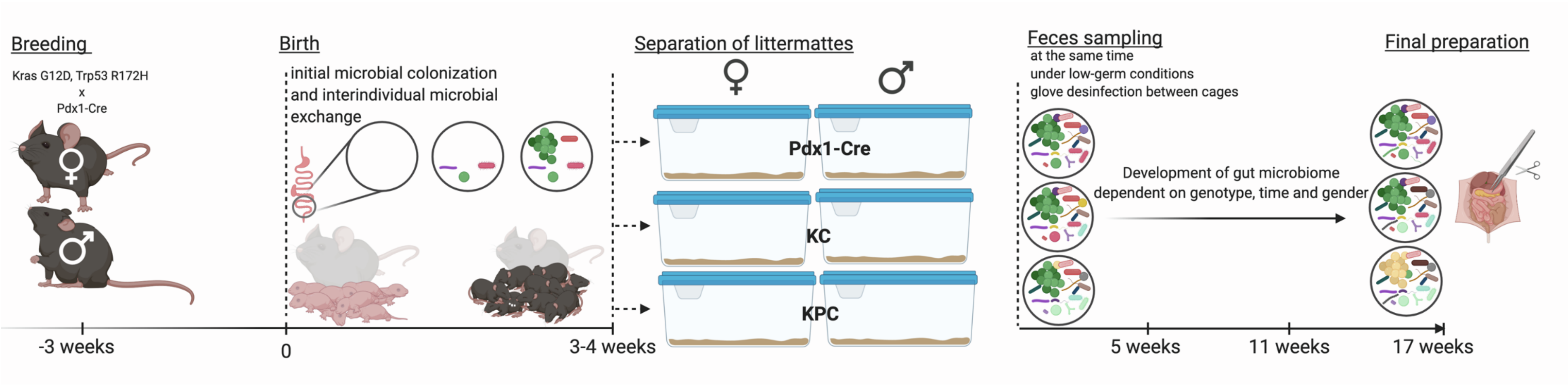

Figure S 6: Experimental design, related to STAR Methods. Pdx1-Cre, KC and KPC mice were genotyped after 2 weeks and selected accordingly. Littermates with the specific genotype (Pdx1-Cre, KC, KPC) were separated from their parents after 3-4 weeks and housed under specific-pathogen-free (SPF)-conditions in genotype- and gender-specific cages with 1-4 animals (mean 1,88, median 2). All interventions were performed under pathogen-free conditions to avoid contamination and feces were collected after 5, 11, and 17 weeks (or the date of death), respectively. The figure was made in ©BioRender – biorender.com.
